# Acknowledgement to Reviewers of *Molecules* in 2018

**DOI:** 10.3390/molecules24010214

**Published:** 2019-01-08

**Authors:** Molecules Editorial Office

**Affiliations:** MDPI, St. Alban-Anlage 66, 4052 Basel, Switzerland

Rigorous peer-review is the corner-stone of high-quality academic publishing. The editorial team greatly appreciates the reviewers who contributed their knowledge and expertise to the journal’s editorial process over the past 12 months. In 2018, a total of 3412 papers were published in the journal, with a median time to first decision of 12 days and a median time to publication of 31 days. The editors would like to express their sincere gratitude to the following reviewers for their cooperation and dedication in 2018:
Aav, RiinaLiu, Xiaoxi (USA)Abadal, SergiLiu, XifengAbarbri, MohamedLiu, XueweiAbashev, George G.Liu, YanbiaoAbbamondi, Gennaro RobertoLiu, YangAbbate, SergioLiu, YongqiangAbbott, David H.Liu, YongqingAbdalla, Maher Y.Liu, YuAbdeeva, A. I.Liu, Yuan-ShuaiAbdel-Daim, MohamedLiu, YujunAbdelwahed, SamehLiu, YunlingAbdulkader, FernandoLiu, ZhichaoAbenavoli, LudovicoLiu, ZhiqiuAbeywardana, TharindumalaLiu, ZhixiaAbraham, Wolf-RainerLivaniou, EvangeliaAbuillan, WasimLivesey, GeoffreyAbul-Haija, YousefLizardi-Mendoza, JaimeAbu-Reidah, Ibrahim M.Llop, JordiAbu-Romman, SaeidLlorens, EugenioAbuzaid, AhmedLloyd, MatthewAccardo, AntonellaLo Meo, PaoloAcero, NuriaLo Scalzo, RobertoAchaerandio Puente, Maria IsabelLo, Kai-YinAcharya, PriyamvadaLoboda, AgnieszkaAchelle, SylvainLocatelli, ClinioAcherar, SamirLocatelli, MarcelloAchilias, Dimitris S.Lodyga-Chruscinska, ElżbietaAchour, BrahimLognay, GeorgesAcosta Contreras, Florentina NiurisLoh, Xian JunAcquah, Gifty E.Lohezic-Le Devehat, FrançoiseAcuña-Castroviejo, DaríoLohman, JeremyAdachi, NaoyaLohning, AnnaAdam, VojtěchLojou, ElisabethAdamcik, JozefLombó, FelipeAdamczyk, BartoszLondon, EdraAdamczyk-Woźniak, AgnieszkaLong, Eric C.Adams, James DavidLongo, RoccoAddepalli, BalasubrahmanyamLongstaffe, James G.Adhikari, Khem BahadurLönnberg, HarriAdhikari, UpendraLopes, Pedro E MAdriana, Estrada-BernalLópez Fernández, HugoAdrover, AlessandraLopez Rodilla, Jesus M.Afantitis, Antreas (Cyprus)Lopez, CristobalAfantitis, Antreas (Greece)López, María L.Afarinkia, KamyarLopez-Escamez, Jose A.Afonin, Andrei V.López-García, JorgeAfonin, KirillLópez-Santos, CarmenAfzal, AdeelLopez-Viñas, EduardoAgamennone, MariangelaLoppi, SteffanoAgarwal, SumitLorberboum-Galski, HayaAgarwal, VinayakLorenz, PeterAgatemor, ChristianLorenzo Abad, María EncarnaciónAgeitos, Jose ManuelLoretz, BrigittaAgliari, ElenaLosada-Perez, PatriciaAgnieszka, SzopaLotina-Hennsen, BlasAgostino, MarkLotti, MarinaAgrawal, PrashansaLou, HongXiangAgrawal, SiddarthLou, SongAgriopoulou, SofiaLouarn, EssylltAguilar, CarmeLoureiro, Jose MiguelAguilar, EnriqueLourenço, AnaAgustin, DominiqueLouro, RicardoAgyei, DominicLovell, JonathanAhmad, ZeeshanLoveridge, E. JoelAhmadi-Afzadi, MasoudLoveridge, JoelAhmed, AlauddinLovrecic, MercedesAhmed, MaryaLowary, ToddAhmed, SaharLozano Ojalvo, DanielAhn, HeekwonLu, Chun-PingAhn, Ji-YoungLu, Hsu-FengAhvazi, BehzadLu, HuasongAiba, YuichiroLu, JunAich, NirupamLu, Mei-ChinAiello, FrancescaLu, QiAigbirhio, Franklin I.Lu, ShenzhouAimanianda, VishukumarLu, WeiyueAires, AlfredoLu, YiAitken, R. AlanLu, ZhanpingAivaras Kareiva, HabilLu, ZhongbingAkamatsu, MikiLubal, PřemyslAkanbi, TaiwoLuber, SandraAkens, MargareteLubin-Germain, NadègeAkitsu, TakashiroLuca, OanaAkk, GustavLucaciu, Constantin MihaiAlabugin, Igor V.Lucarini, MassimoAlaimo, SalvatoreLucas, KurtAlam, Md BadrulLucas, XavierAlam, Mohammad AbrarLucassen, PaulAlam, Mohammad ParvezLucena, RafaelAlasbahi, BandarLuchinat, EnricoAlashi, Adeola MLucia Tornesello, AnnaAlbanese, DomenicoLucini, LuigiAlbergamo, AmbroginaLuck, RudyAlbergante, LucaŁuczak, JustynaAlbericio, FernandoLudwiczuk, AgnieszkaAlberto, RogerLugo-Cervantes, EugeniaAlbiniak, PhilipLui, Edmund Man KingAlbo, JonathanLuís, ÂngeloAlbuquerque, MagalyLuis, Valencia CabreraAldabbagh, FawazŁukajtis, RafałAlder, JaneŁukasik, RafałAlegre, CinthiaLukaszkowicz, KrzysztofAlexander, Bruce DLukinavicius, GrazvydasAlexis, FrankLukyanov, PavelAlexopoulos, AthanassiosLuliński, SergiuszAlfano, AlbertoLung, Ming-YeouAlfano, DanielaLuo, LynaAl-Fatimi, MohamedLuo, MinkuiAlferink, JudithLuo, RongcongAlhazmi, Hassan A.Luo, ZishengAl-Horani, RamiLuo, ZonghuaAlizadeh, HosseinLuque, F. JavierAlkorta, IbonLuque, RafaelAllain, FabriceLuscombe, ChristineAllais, FlorentLuthra, AmitAllinquant, BernadetteLütticken, RudolfAllsopp, Luke PLutz, MartinAlmajano, María PilarLuukkonen, TeroAlmeida, HenriqueLuxen, AndréAlmendros, GonzaloLykakis, IoannisAlmoazen, HassanLykidis, CharalamposAlonso, Diego A.Lyu, HuiAlonso, LeocadioLyutakov, OleksiyAlonso, Rosa MariaM. Keseru, GyorgyAl-Rimawi, FuadMa, ChaoAltemimi, AmmarMa, CongAltieri, FabioMa, GuojiaAltmann, FriedrichMa, LiangAlvarez, CarlosMa, QinÁlvarez, JoaquínMa, TianyiAlvarez, LauraMa, YongÁlvarez-Fernández, M. AntoniaMa, ZexuAlvarez-Idaboy, Juan RaùlMa, ZhikunAlvarez-Suarez, JoséMaak, SteffenAlves, Carlos RobertoMaász, GáborAlves, ElianaMabou Tagne, AlexAlves, Harley Da SilvaMaccallini, CristinaAlves, Maria JoséMacchi, BeatriceAlves, Vítor H.Macchiarulo, AntonioAmano, MasafumiMaccione, EliasAmarowicz, RyszardMacernis, MindaugasAmato, JussaraMachetti, FabrizioAmato, RosarioMacia, AngelaAmelin, Vasiliy G.Mackereth, CameronAmengual, JaumeMaçôas, ErmelindaAmgoune, AbderrahmaneMacone, AlbertoAmi, DilettaMacwan, IsaacAmigo-Benavent, MiryamMączka, WandaAmii, HidekiMadan, VanesaAmin, M. JunaidMaddau, LuciaAmirkhani, MasoumeMadesis, PanagiotisAmmendola, RosarioMadine, JillianAmodeo, PietroMadrid, AlejandroAmorati, RiccardoMadsen, Andreas S.Amorós, AsunciónMady, Mohamed F.Amrutkar, ManojMadzhidov, TimurAmsharov, KonstantinMaeda, TomokiAn, YuMaeng, Han-JooAnadón, ArturoMafra, Marcos RogérioAnand, VijayMafu, SibongileAnas, Andrea RoxanneMagain, NicolasAncuceanu, RobertMagalhães, Júlia M.C.S.Ancuceanu, Robert ViorelMagana, Luis FernandoAndersson, Håkan SMagata, YasuhiroAndjelkovic, MirjanaMaGee, DavidAndo, KaoriMaggioni, DanielaAndolfi, AnnaMagiorkinis, EmmanouilAndrade, FernandoMagnabosco, GiuliaAndrade, IsabelMagnaldo, ThierryAndrade, Paula BMagni, PaoloAndrea, PenoniMagnus, MarcinAndreana, PeterMagri, David C.Andrés-Sánchez, SantiagoMagriotis, Plato A.Andrésson, Ólafur S.Mahal, KatharinaAndrianasolo, EricMahavadi, PoornimaAndrić, FilipMaher, PamelaAndruszkiewicz, RyszardMahimainathan, LeninAnfossi, LauraMahmoud, Alaa El DinAngelico, RuggeroMahmudov, Kamran T.Angelova, Violina T.Mahrwald, RainerAngilella, Giuseppe G. N.Maican, EdmondAngiolella, LetiziaMaicas, SergiAnna, SzakalMainero Rocca, LuciaAnnor, GeorgeMaisch, TimAnraku, MakotoMajewski, LeszekAntal, DianaMajireck, MaxAntanasijević, JelenaMajkowska-Skrobek, GrażynaAnticó, EnriquetaMajtan, JurajAntoni, GunnarMajumder, KaustavAnuta, ValentinaMakarska-Bialokoz, MagdalenaAnzai, Jun-ichiMakena, MonishAnzenbacher, PavelMaker, GarthAoki, DanMalaguarnera, GiuliaApartsin, EvgenyMalawska, BarbaraApetrei, ConstantinMalde, AlpeshAprea, EugenioMalekigorji, MaryamAprodu, IulianaMaleknia, Simin DAquaro, StefanoMalla, SpundanaAragó, JuanMallavia, RicardoArai, ToshiroMallipeddi, Prema LathaAraki, KazuoMalmberg, PerArambula, JonathanMalmström, LarsArancibia, RodrigoMaloberti, AlessandroArango, Rachel A.Malterud, KarlAraniciu, CătălinMamane, VictorArano, YasushiManabe, KeiArcadi, AntonioManavalan, BalachandranArchibald, SteveManca, Maria LetiziaArguelles, SandroMancera, Ricardo L.Arion, VladimirMancianti, FrancescaArita, MasanoriMancini, InesArmentrout, Peter B.Mancini, RockArnao, Marino BMancini, SimoneArnason, JohnManciu, FeliciaArrachart, GuilhemManconi, MariaArroo, RandolphMancuso, RaffaellaArroyo, NetzahualcóyotlMandadi, KranthiArteaga, Jesús F.Mandal, DebashisArtetxe, BeñatMandal, SoumyajitArtola, MartaMandalari, GiusyArtyszak, ArkadiuszMandic, ZoranArvapally, Ravi KumarMandity, IstvanAsai, DaisukeMandoli, AlessandroAsakawa, YoshinoriMandrone, ManuelaAsamizu, ShumpeiManfredini, StefanoAscenzi, PaoloMangalagiu, IonelAsfin, Ruslan E.Manian, Avinash P.Ashley, JonManjunath, ManuboluAskes, SvenMankin, Richard W.Asmontas, SteponasManley, SulianaAspatwar, AshokMann, FrancisAssari, ShervinManner, SuviAssfeld, XavierMano, YujiÅstrand, AlexanderManoury, EricAtanasova-Penichon, VesselaManríquez-Torres, José De JesúsAtef, EmanManthey, John A.Atobe, MasakazuMantle, PeterAttanasio, ChiaraMantzioris, EvangelineAubert, EmmanuelMantzouridou, FaniAudette, Gerald FManzano, Blanca R.Auvynet, ConstanceManzhos, SergeiAvato, PinarosaManzone, MarcoAvci, FikriMao, Meng (Australia)Avdeeva, Varvara V.Mao, Meng (USA)Averin, Alexei D.Mao, ZongwanAviles, Francesc XavierMarangon, MatteoAvin, VijayMarasco, DanielaAwale, SureshMarasini, NirmalAyala-Zavala, J. F.Marazzi, MarcoAzab, AbdullatifMarc, DanielAziz, AtharMarceau, FrançoisAzman, SametMarcelis, LionelB. Luwor, RodneyMarchal, RichardBaba, HiromiMarchesan, SilviaBabaev, EugeneMarcin Henryk, StruszczykBabii, OlegMarco, FranciscoBaby, AndréMarco-Contelles, JoséBacelar, EuniceMarek, AntoninBach, Duc-HiepMarestin, CatherineBack, KyoungwhanMargherita, MaioliBacolla, AlbinoMari, MicheleBácskay, IldikóMaria, CorosBacso, ZsoltMaria, Diaconeasa ZoriţaBączek, KatarzynaMarichev, KostiantynBączek, TomaszMarin, LuminitaBadawi, MohamedMarín, MercedesBaddeley, DavidMarincs, FerencBado, IgorMarini, SimoneBae, JiyoungMarino, TizianaBae, Ki HyunMarjanović, MarkoBaek, Hyung HeeMárki, ÁrpádBaek, Kwang-HyunMarkiewicz, Wojciech T.Baek, Nam-InMarkkanen, EnniBaeza, AlejandroMarkopoulou, OlgaBagdziunas, GintautasMarla, SandeepBahn, AndrewMarqués Villavecchia, Ana M.Bai, FangMarra, AlbertoBai, MingfengMarrelli, MariangelaBai, RenrenMarrocchi, AssuntaBai, ShiqiangMarrone, AlessandroBaier, AndreaMarschner, ChristophBailly, FabriceMarszalek, KrystianBajaj, SumitMarta, Ferreiro-GonzálezBajpai, RichaMartelli, Pier LuigiBak, AndrzejMartí, SergioBak, IstvanMartín, Antonio E.Bakare, OladapoMartin, FinianBakillah, AhmedMartin, JessicaBaksh, ShairazMartin, Jose MiguelBakunina, IrinaMartin, LaetitiaBalan, VenkateshMartin, NathanielBalasubramaniam, MeenakshisundaramMartín-Antonio, BeatrizBalasubramaniam, SivaramanMartín-Aragón, SagrarioBalbach, JochenMartinelli, AdrianoBalcerek, MariaMartinello, MariannaBaldisserotto, AnnaMartínez Vázquez, Rafael FernandoBálint, ErikaMartínez, AlbertoBaliraine, FrederickMartinez, AnaBallard, NicholasMartínez, AnaBallmer-Hofer, KurtMartínez, AntonioBally, JuliaMartinez, FlemingBalogh, György TiborMartínez, SidoniaBaltakys, KęstutisMartínez-Ávila, GuillermoBalzer, FrankMartinez-del-Pozo, AlvaroBanach, MarcinMartinez-Gil, LuisBanchero, MauroMartínez-Gómez, PedroBandar, Jeffrey S.Martinez-Lostao, LuisBandyopadhyay, DebasishMartin-Fontecha, AlfonsoBandyopadhyay, SulalitMartín-Hernández, DavidBanerjee, AditiMartini, ClaudiaBanerjee, KalpitaMartini, DanielaBanerjee, SudipMartínková, LudmilaBaniahmad, AriaMartino, MarcoBankina, BirutaMartín-Peláez, SandraBankova, VassyaMartín-Ramos, PabloBanning, AntjeMartins, Alice M.Banoub, JosephMartins, Ian JamesBanskota, Arjun H.Martins, Luísa MargaridaBansode, AtulMartins, Maria RosarioBanti, Christina N.Martins, NatáliaBantreil, XavierMartins, SoniaBao, AndeMartins-Lopes, PaulaBao, YinyinMarto, JoanaBao, Yong-PingMartoïa, FlorianBarakat, AssemMartorana, AlessandroBaraldi, Pier GiovanniMaru, Marcia M.Baranski, RafalMaruf, Abdullah AlBarattucci, AnnaMaruyama, IchiroBarba, Anna A.Maruyama, KeiBarba, Francisco J.Marverti, GaetanoBarbaric, MonikaMaryasin, BorisBarbero, Gerardo FernándezMarycz, KrzysztofBarbieri, AntonioMarzaro, GiovanniBarbieri, PierluigiMarzo, TizianoBarbillon, GrégoryMarzocco, StefaniaBarchi, Joseph J.Masek, AnnaBarczyński, PiotrMashima, RyuichiBardají, EduardMasi, MarcoBardelang, DavidMasino, FrancescaBarendt, TimothyMaslov, Mikhail A.Barišić, LidijaMasłyk, MaciejBarja-Fidalgo, ChristinaMassa, AntonioBarker, Bridget M.Massimi, IsabellaBar-Kohany, TaliMassiot, GeorgesBarlocco, DanielaMásson, MárBarman, ApurbaMassoud, SalahBarnych, BogdanMastanjević, KristinaBaron, MarcoMastinu, AndreaBarra, Guilherme M.O.Mastrangelo, Anna M.Barradas, Thaís NogueiraMasu, HyumaBarreira, João C.M.Masuda, KiyoshiBarrero, Carlos AMasui, KentaBarreto, GuillermoMasullo, MariorosarioBarreto, Maria Do CarmoMata, ErnestoBarros, AntonioMata, Jose A.Barros, Pedro M.Mata, RachelBarry, CorneliusMata, RicardoBarry-Ryan, CatherineMatczak-Jon, EwaBar-Shavit, ZviMateo, CesarBartelt, AlexanderMateos, RaquelBartocci, PietroMaterska, MałgorzataBarton, Larry L.Mathé, Ewy A.Bartoněk, DaliborMatiadis, DimitrisBartosz, GrzegorzMatilla, MiguelBartoszewski, RafalMatilla-Hernández, AntonioBartzas, GeorgiosMatos Lale, JuanBaryshnikov, GlibMatosiuk, DariuszBasak, Ajit K.Matouskova, PetraBasak, SujitMatsubara, KoukiBasile, TeodoraMatsuda, FumioBasini, GiuseppinaMatsufuji, HiroshiBasit, AbdulMatsugo, SeiichiBassin, PaulMatsui, DaisukeBassoli, AngelaMatsumoto, ArimasaBasu, SwarajMatsumoto, Ken'ichiroBatail, PatrickMatsumoto, KozoBatista, IrineuMatsumoto, ShojiBattistelli, CeciliaMatsumoto, YasuhikoBatty, Kevin T.Matsuo, TakashiBatule, Bhagwan SahebraoMatsuo, YosukeBauer, EikeMatsuo, YukikoBauza, AntonioMatsuura, BunzoBavaro, Simona LuciaMatsuura, KazunoriBavaro, TeodoraMatta, CherifBayer, PeterMattevi, AndreaBayón, PauMattoo, AutarBaz Etchebarne, MarianaMatts, RobertBazylińska, UrszulaMatwijczuk, ArkadiuszBazzicalupi, CarlaMauricio, CuellarBeata, Kalska-SzostkoMavromoustakos, ThomasBeaudoin, DanielMayhan, William GBeaudry, ChristopherMazerska, ZofiaBębenek, EwaMazur-Bialy, Agnieszka IrenaBeberok, ArturMazurek, SylwesterBechthold, AndreasMazzoni, LucaBecker, Therese M.Mazzoni, RitaBedia, CarmenMazzotti, FabioBedini, EmilianoMcCarthy, ElizabethBednar, PetrMcCarthy, PumtiwittBedos-Belval, FlorenceMcDaniel, JonathanBeeton, NickMcdougal, Owen M.Begley, DavidMcDougall, CarmelBeharry, Kay D.McFeeters, RobertBehrends, SoenkeMcGlinchey, Michael J.Beilby, MaryMcGonigal, Paul R.Beis, DimitrisMcGowan, EileenBekhit, Alaa El-Din A.McGowan, SheenaBekiaris, GeorgiosMcKeague, MaureenBel’skaya, Lyudmila V.McKee, Lauren S.Belancio, VictoriaMcLaughlin, MarkBeld, JorisMcMorran, DavidBeletskaya, Irina P.McMullin, David R.Belfrage, Anna KarinMeadus, William JonBell, KarenMeana-Pañeda, RubénBell, Thomas W.Medarde, ManuelBella, FedericoMedeiros, Adriane Bianchi PedroniBellés, José MaríaMedellin-Castillo, NahumBello, ClaudiaMedici, SerenellaBelmont, PhilippeMedina, MilagrosBeloqui, AnaMedina-Vera, MyriamBełtowski, JerzyMedrano, HipolitoBen Mahmoud, LobnaMedvidović-Kosanović, MartinaBenati, DanielaMeegan, MaryBencsik, TimeaMeenashisundaram, Ganesh KumarBendas, GerdMegighian, AramBenedec, DanielaMegyeri, KlaraBenedetti, MicheleMehaffy, CarolinaBeniah, GoliathMehbub, Mohammad FerdousBenito, AntoniMehdi, AhmadBenito, José ManuelMehta, Akul Y.Benito, Juan M.Meier-Menches, Samuel M.Bennet, AndrewMeijsing, SebastiaanBenseny-Cases, NuriaMeisrimler, Claudia-NicoleBenso, BrunaMekinić, Ivana GeneralićBenyoucef, AbdelghaniMele, AndreaBenzeroual, Kenza E.Melguizo-Guijarro, ManuelBerdonosov, PeterMelini, ValentinaBerenjian, AydinMella Raipan, JaimeBerenyiova, AndreaMellbye, Brett L.Beresford, TomMelo, AndréBeretta, GiangiacomoMelzig, Matthias F.Beretta, GiovanniMENA, PedroBergman, JanMenabue, LediBerhow, MarkMencherini, TeresaBerlin, K. DarrellMendes, AdrianoBerlowska, JoannaMendes, Livia P.Bermudez, MarcelMéndez, LucíaBernal, PatriciaMendez-Sanchez, NahunBernard, Marek K.Mendiola, JoséBernardi, LucaMendive-Tapia, DavidBernardini, GiovanniMendonca, AntonioBernini, RobertaMendoza-Nuñez, Victor ManuelBernstock, Joshua D.Meneghetti, FiorellaBerrada, HoudaMenet, Marie ClaudeBerreau, Lisa M.Menezes, Irwin Rose AlencarBerrocal, José AugustoMenezes, JoséBerruyer, RomainMenezes, ReginaBerski, WiktorMenna, MarialuisaBert, NikolayMenon, JyothiBerthelsen, RagnaMercer, JasonBerthold-Pluta, AnnaMerfort, IrmgardBerti, FedericoMergler, StefanBertin, Matthew J.Merino, GraciaBerton, PaulaMernyák, ErzsébetBertran, JoanMerten, ChristianBertucci, MichaelMertens, FredrikBesalú, EmiliMeschwitz, SusanBescond, JocelynMesgar, MiladBesson, ThierryMessali, MouslimBetancourt, TaniaMessyasz, BeataBethanis, KostasMester, Patrick-JulianBettuzzi, SaverioMetzger, Robert M.Beutel, SMetzinger, LaurentBeversdorf, DavidMeyerholz, David K.Bevilacqua, ArturoMézes, MiklósBeyeh, Ngong KodiahMező, GáborBezirtzoglou, EugénieMi, LanBhang, Suk HoMi, XiaochengBharadwaj, VivekMiao, TianxinBhattacharya, DebswapnaMichalska, AnnaBhullar, Rajinder P.Michalski, RajmundBiałoń, MariettaMichaud, PhilippeBian, YongzhongMicheau, OlivierBianchi, AntonioMichel, Martin C.Biancolillo, AlessandraMichel, PiotrBickhart, Derek M.Micheletti, GabrieleBiedermann, DavidMichels, Alexander J.Bielenica, AnnaMichiho, ItoBiernasiuk, AnnaMicillo, RaffaellaBiesaga, MagdalenaMickelson, Alan R.Bijak, MichałMiękus, NataliaBikiaris, DimitriosMiele, Adriana E.Bilewicz, AleksanderMielenz, ManfredBilitewski, UrsulaMiguel, MariaBill, HawkinsMiki, KojiBillard, ThierryMiki, YasuhiroBilyachenko, Alexey N.Mikołajczyk-Bator, KatarzynaBimbo, Luis M.Mikstacka, RenataBingol, KeremMikulic-Petkovsek, MajaBinzel, Daniel W.Mikulskis, PauliusBiondi, ElisaMikuš, PeterBiot, ChristopheMilano, GiuseppinaBirch, JohnMilas, Haralampos N.Birkemeyer, ClaudiaMilella, LuigiBirringer, MarcMiles, Wayne OwenBishop, ReidMilia, EgleBisio, AlessandraMilledge, John J.Bisio, AntonellaMiller, John H.Bisoyi, Hari KrishnaMiller, JustinBisson, JonathanMillet, OscarBisti, SilviaMillo, EnricoBisz, ElwiraMimaki, YoshihiroBlack, DavidMinami, YasunoriBlackburn, JessicaMinamoto, ToshinariBlackwell, BarbaraMinato, Ken-ichiroBlair, PatriciaMinato, MakotoBlanco-López, María CarmenMinceva, MirjanaBlando, FedericaMine, YuichiBlanton, CynthiaMinehan, Thomas G.Blasiak, JanuszMinervini, GiovanniBłaszczak, WiolettaMinetti, GiampaoloBlenda, AnnaMinjares-Fuentes, Jose RafaelBlériot, YvesMinkiewicz, PiotrBloom, JesseMinnaar, PhillipBloor, StephenMinoru, HayashiBo', Cristian DelMintcheva, NeliBo, ZhenyuMinucci, SergioBoard, MaryMinutolo, FilippoBobis, OtiliaMirali, MahlaBoccard, JulienMiranda, CristobalBocchinfuso, GianfrancoMiranda, Jose M.Boccia, Antonella CaterinaMiranda, KatrinaBock, ThomasMiras-Avalos, Jose ManuelBoddy, Christopher N.Mironescu, MonicaBode, Robert F.Mirossay, LadislavBodenstine, ThomasMirzayans, RazmikBoeck, Lorenz RMishin, Alexander S.Boeriu, CarmenMishra, Geetesh KumarBoettcher, ShannonMishra, NigamBoga, CarlaMishra, Rupesh K.Bogdan, CatalinaMishra, SandhyaBogdanowicz, Krzysztof ArturMishra, SasmitaBøgwald, JarlMisicka, AleksandraBöhm, VolkerMisurcova, LadislavaBöhmer, FrankMita, Damiano GustavoBohn, TorstenMita, GiovanniBoichuk, SergeiMitakou, SofiaBojić, MirzaMitchell, JohnBokach, NadezhdaMitev, Pavlin D.Bollyky, PaulMitryukovskiy, SergeyBolognini, DanieleMitsudo, KoichiBölükbas, DenizMittal, GayatriBombard, SophieMiura, ToshikoBombarely, AurelianoMiyabe, HidetoBommagani, ShbanbabuMiyagi, AtsukoBon, VolodymyrMiyamoto, HirotakaBonaccio, MarialauraMiyamoto, KojiBonamore, AlessandraMiyata, YasuyoshiBondon, ArnaudMiyata, YoshihikoBonetta, SaraMiyazato, HironariBongarzone, SalvatoreMizielińska, MałgorzataBonifácio, VascoMizuno, Cassia SBonikowski, RadosławMizuno, TakayukiBonnet, Pierre-AntoineMizuta, KentaroBonomo, Robert AMladěnka, PřemyslBooth, BrianMladenovic, MilanBora-Singhal, NamrataMlejnek, PetrBorawska-Dziadkiewicz, JustynaMnif, WissemBorbás, AnikóMobley, JustinBorcan, FlorinMocan, AndreiBordenave-Juchereau, StéphanieMochona, BereketBordons, AlbertMockaitis, GustavoBorges, AnabelaModahl, CassandraBorges, FilipeModec, BarbaraBorges, Keyller BastosModhiran, NaphakBorges, RicardoModugno, FrancescaBorghini, AndreaModukuri, RamkumarBorin, Thaiz FerrazMoen, ErikaBormashenko, EdwardMogielnicki, AndrzejBoros, LaszloMohamed, SharmarkeBorota, AnaMohammadiarani, HosseinBorovkov, VictorMohammed, YousufBorst, Jan WillemMohankumar, KumaravelBoschi, AlessandraMohanram, HariniBose, DebojitMohanta, TapanBose, SayantanMoine, LaurenceBoselli, EmanueleMoineau-Chane-Ching, Kathleen I.Bosi, SaraMojsoska, BiljanaBosland, Maarten C.Mojzych, MariuszBosse, Jens B.Mok, Daniel Kam-WahBotelho, Monica C.Moliner Martinez, YolandaBotre, FrancescoMollace, VincenzoBouaziz, SergeMollapour, MehdiBouckaert, JulieMollica, AdrianoBoulton, RogerMolnar, MajaBouquillon, SandrineMolognoni, LucianoBour, PetrMoloney, Mark G.Bourguet-Kondracki, Marie-LiseMolugu, Sudheer KumarBoušová, IvaMomken, ImanBoutin, JeanMonaghan, DanielBoutin, Jean A.Monari, AntonioBowden, GregoryMonchaud, DavidBowling, HeatherMonclus, MichelBoyarskiy, Vadim P.Mondejar, Maria E.Boysen, ReinhardMonflier, EricBozonet, Stephanie M.Mong, Mei-ChinBožović, MijatMoni, LisaBrábek, JanMonika, Waksmundzka-HajnosBracci, MassimoMoniuszko-Szajwaj, BarbaraBrachet, EtienneMonroe, Mary Beth BrowningBrader, GünterMontag, JudithBradshaw, BenMontalbán, Mercedes G.Braga, AntonioMontaño Priede, José LuisBraga, Guilherme S.Monteil-Rivera, FannyBraga, SusanaMonteleone, StefaniaBraig, SimoneMontenegro, LuciaBrandstetter, HansMontero, OlimpioBrandt, CurtisMontesano, DomenicoBranza-Nichita, NoricaMontesarchio, DanielaBranzoi, FlorinaMontgomery, MagdaleneBraunecker, WadeMontowska, MagdalenaBravo López, Susana BMoody, David EBravo, JeronimoMoon, Byeong CheolBrčić Karačonji, IrenaMoon, Doo KyungBrebu, MihaiMoon, IlsooBrecker, LotharMoon, KyungBreier, AlbertMoore, JohnBreinbauer, RolfMoore, Robert J.Breitenfeld Granadeiro, LuizaMoore, StuartBrem, JurgenMorais, MauricioBren, UrbanMorales, GabrielBrenna, ElisabettaMorales-González, José AntonioBrennan, MarianMorales-Morales, DavidBrenner, RobertMorán, José M.Brestic, MarianMorávková, ZuzanaBrettell, Laura E.Moreau, RegisBrevern, AlexandreMoreira, JoséBreza, MartinMorelli, SabrinaBrichacek, MatthewMoreno, AndrésBriskey, DavidMoreno, Antonio D.Broberg, AndersMoreno, Diego A.Broderick, Tom L.Moreno, MiguelBrooks, PeterMorganti, PierfrancescoBrooks, Tracy A.Mori, BoydBros, MatthiasMori, MattiaBrouwers, Jos F.Mori, SeijiBrown, LindsayMori, ShigekiBrown, Robert J.Morigi, RitaBruckner, ChristianMoriguchi, TakayaBrudzynski, KatrinaMorikawa, ToshioBrullo, ChiaraMorimoto, MasanoriBrun, RetoMorimoto, YumaBrune, WolframMorimura, ShigeruBruni, GiovannaMorita, NaokiBruning, John B.Morkunas, IwonaBrunklaus, GuntherMoro, LoredanaBruno, OlgaMorrill, Louis C.Brunschweiger, AndreasMorris, GordonBrust, PeterMorris, James L.Brustovetsky, NikolayMortier, JérémieBryant, Matthew S.Morton, DavidBrycki, BogumilMorzycki, Jacek W.Brzeska, JoannaMosawy, SaphaBrzeziński, MarekMoschou, Panagiotis N.Brzozowski, ThomasMoscobe, DanilaBubb, Kristen J.Mosquera, MartaBubici, GiovanniMossine, Valeri V.Bubulya, Paula A.Most, JasperBučar, Dejan-KrešimirMostafavi, MehranBucar, FranzMot, AugustinBuchet, ReneMota, Antonio J.Buchner, DavidMota, ManuelBuchowicz, WłodzimierzMouahid, AdilBuchwald, PeterMoura, Maria J.Budnikova, YuliaMourtas, SpyridonBudny, MarcinMourtzinos, IoannisBudryn, GrażynaMovassagh, HesamBudzak, SimonMoyano, AlbertBudzisz, ElżbietaMoyano, LourdesBueno Cavanillas, AuroraMphahlele, Malose JackBueno-Silva, BrunoMrówczyński, RadosławBufo, Sabino AurelioMsaada, KamelBugarin, AlejandroMucha, ArturBuisson, DidierMucsi, ZoltánBukin, OlegMuehlmann, Luis AlexandreBułakowska, AnitaMueller, AnjaBulanin, KirillMueller, Wolf C.Bulgariu, LauraMuggia, LuciaBullon, PedroMuhlack, RichardBultynck, GeertMukaida, NaofumiBun Ng, TziMukherjee, SoumyaBunce, Richard A.Mulas, MaurizioBundgaard, EvaMulet, J. M.Bundle, David R.MULLER, ChristianBunik, VictoriaMüller, KarstenBunt, CraigMuller, Patricia A. J.Bura-Nakić, ElviraMüller, Werner E.G.Burcul, FrankoMulloy, BarbaraBurger, MichaelMun, JiyoungBurghardt, KyleMuñiz Rodriguez, PilarBurkard, MarkusMunteanu, Bogdanel SilvestruBurns, Duncan ThorburnMuramoto, KojiBurpo, Fred J.Murata, JunBurrola-Aguilar, CristinaMurata, ToshihiroBury, WojciechMuresan, VladBusch, HaukeMurillo Tovar, Mario AlfonsoBuschbeck, MarcusMurkovic, MichaelBuß, OliverMurotomi, KazutoshiBustamante, PacoMurphree, S. ShaunBustos, Diego MartinMurphy, CormacButenschön, HolgerMurphy, NeilButler, MarkMurray, JaneButterworth, Peter J.Murugaiyan, JayaseelanBuxton, DenisMurugan, N. ArulBuzina, WalterMuruganandan, ShanmugamByrne, BernadetteMuschiol, JanByrne, HughMusgrave, IanBystrowska, BeataMusiol, RobertBywater, Robert P.Musmarra, DinoBzducha-Wróbel, AnnaMusso, IoanaCaballero, JulioMuszyńska, BożenaCaballero-Garcia, BeatrizMuthukumar, ThangaveluCabral, CéliaMuthuramu, IlayarajaCabral, HoracioMutin, HubertCabrales, LuisMuziol, Tadeusz MikolajCabrele, ChiaraMwinyi, JessicaCabrita, Ana Rita JordãoMyasoedova, VeronikaCacciola, FrancescoMyers, StephenCáceres González, Francisco F. Núñez DeMyllykallio, HannuCadamuro, MassimilianoMyrianthopoulos, VassiliosCádiz-Gurrea, María LuzMyśliwiec, HannaCaeiro, MariaMystkowska, JoannaCaffrey, PatrickMystrioti, C.Cafiero, MauricioN. Setzer, WilliamCahlíková, LucieNa, Dong HeeCahoon, Aubrey BruceNabissi, MassimoCai, FengNabok, AlexeiCai, ZhengxinNaccarato, AttilioCaiazzo, ElisabettaNaesens, LieveCailliez, FabienNagai, KouheiCairrão, ElisaNagai, YasukiCais-Sokolińska, DorotaNagai, YoshinoriCalani, LucaNagaraj, ChandranCalcabrini, CinziaNagasawa, KazuoCalcutt, MichaelNagatsugi, FumiCalderon, Christopher P.Nagel, RaimundCalderone, Christopher T.Nagy, IstvanCaligiuri, IsabellaNaha, Pratap C.Calingacion, MariafeNahid, Abdullah-AlCalokerinos, Antony C.Nair, RemyaCalvano, Cosima DamianaNair, VimalCalvete, Juan J.Nairz, ManfredCalvete, MarioNajlah, MohammadCamacho, M. EncarnaciónNak Cheon, JeongCamacho-Corona, M. R.Nakagawa-izumi, AkikoCâmara, José SousaNakai, YumiCamele, IppolitoNakajima, MasahiroCamerlingo, CarloNakajima, MasatoshiCaminade, Anne-MarieNakamura, ItaruCampaña, Araceli G.Nakanishi, WakaCampana, RaffaellaNakano, KojiCampanella, ClaudiaNakao, YoichiCampardelli, RobertaNakashima, SouichiCampbell, ChristopherNakata, NorioCampbell, Fiona M.Nakata, SatoshiCampbell, LeeNakatani, YoshihikoCampestre, CristinaNakatsu, KanjiCampos Da Rosa, Joaquim MNallasamy, PalanisamyCampos-Martínez, J.Nam, Joo-WonCampuzano, SusanaNam, Ju-OckCanals, FrancescNam, KwanghoCancemi, PatriziaNam, Sang-HoCanetta, ElisabettaNam, Sang-JipCanini, AntonellaNamasivayam, VigneshwaranCao, XianNamgaladze, DmitryCao, ZheNamiesnik, JacekCaparrotta, StefaniaNamieśnik, JacekCapasso, RaffaeleNanbu, ShinkohCapobianco, AmedeoNandha Premnath, PadmavathyCapobianco, GiuseppeNandi, Saikat (Sweden)Cappariello, AlfredoNandi, Saikat (USA)Cappellini, FrancescaNanev, ChristoCapriati, VitoNanni, LorisCaputo, Gregory A.Nano, AdelaCarbonell-Barrachina, Angel A.Narayan, PrakashCárdenas, SoledadNarayanan, GaneshCardenia, VladimiroNardi, MonicaCardia, Maria CristinaNardi, SerenellaCardiano, PaolaNardi, TizianaCardinale, JensNariai, TadashiCardinale, MassimilianoNarita, AkihiroCardinali, AlessandraNarsimhan, GanesanCardoso, HéliaNarváez, ManuelCardoso, SusanaNasef, Noha AhmedCardoso, Susana M.Nastasa, CristinaCardullo, NunzioNatadra Tabudravu, JiojiCarella, Angelo MNath, ArijitCarillo, PetroniaNatskoulis, PantelisCarlberg, CarstenNaughton, DeclanCarlisi, DanielaNaumann, StefanCarmona-Ribeiro, Ana M.Naumova, Ella A.Carocho, MarcioNaumovski, NenadCarotti, AndreaNavacchia, Maria LuisaCarotti, AngeloNavarrete-Vazquez, GabrielCarpene, ChristianNavarro, RodrigoCarqueijeiro, InêsNaviglio, DanieleCarradori, SimoneNawaz, MuhammadCarrano, Carl J.Nawirska-Olszańska, AgnieszkaCarrasco, SergioNaya, FranciscoCarrero, Jose AntonioNazarov, Andrej P.Carretti, EmilianoNazarski, Ryszard B.Carta, FabrizioNazzaro, FilomenaCarter, NicolaNdieyira, JosephCarter, WayneNeal, LukeCarullo, GabrieleNedachi, TakuCaruso, GiuseppeNedialkov, Paraskev TodorovCarvalho Pimenta, DanielNeelamegham, SriramCarvalho, Nakédia M.F.Nees, MatthiasCarvalho, PedroNegrete, GeorgeCasalino, ElisabettaNehira, TatsuoCasapullo, Agostino Nehmé, ReineCasas Ferreira, Ana MaríaNeilson, AndrewCasassa, L. FedericoNejad, MojganCasassa, Luis FedericoNemzer, BorisCascioferro, StellaNencka, RadimCascione, MariafrancescaNeng, NunoCaselli, AlessandroNepal, MadhavCaseri, WalterNeri, PlacidoCastaneda, Carlos H.Nesterov, DmytroCastan-Laurell, IsabelleNesterova, Irina V.Castellucci, SoniaNetto-Ferreira, Jose CarlosCastiglione, FrancaNeugart, SusanneCastñeiras, AlfonsoNeuhaus, JochenCastro, MariaNeumann, HeinzCatani, MartinaNeumann, TerrenceCatanzano, OvidioNeunert, GrazynaCatizzone, EnricoNevárez-Moorillón, Guadalupe VirginiaCattaneo, FabioNeves, Adriana CunhaCatto, MarcoNewman, David J.Catucci, LuciaNewman, StephenCauli, OmarNg, Tzi BunCava, ClaudiaNgai, Ming-YuCavaleiro, CarlosNgai, ToCefalas, A.C.Ngamcherdtrakul, WorapolCelaya-Padilla, José M.Nguyen, Thanh BinhCeleiro, MaríaNguyen, TrieuCeliński, KonradNi, DalongCellamare, SaverioNichols, DavidČėnas, NarimantasNicolás, Francisco E.Cendrowski, AndrzejNicoletti, FerdinandoCeranowicz, PiotrNicoletti, RosarioCerchia, LauraNicosia, AldoČerný, JiříNiederman, Robert A.Cerón-Carrasco, José PedroNiedziela, TomaszČeřovský, VáclavNiehaus, Eva-MariaCerra, BrunoNiehaus, TomCeruti, StefaniaNielsen, LarsCesa, StefaniaNielsen, Line HagnerCha, ChaenyungNielsen, Vance G.Chacón, PabloNieman, David C.Chae, HeeyoungNieminen, KaarloChai, Jeng-DaNiemirowicz-Laskowska, KatarzynaChai, Tian FengNiidome, TakuroChaimbault, PatrickNikas, Spyros P.Chakraborty, RajaNikel, Pablo IvanChamakura, KarthikNikolaev, ViacheslavChambers IV, EdgarNikolakaki, EleniChambon, ChristopheNikolakakis, IoannisChamorro-Posada, PedroNikolakopoulou, Angeliki M.Chamrád, IvoNikolopoulos, D.Chan, Ben C. L.Nilghaz, AzadehChan, Yi-TsuNilsson, AkeChan, Yu-YiNilsson, HenrikChandaka, Giri KumarNimalaratne, ChamilaChandasana, HardikNishi, KosukeChang, Che-ChienNishida, TakehiroChang, Chia-ChenNishikitani, YoshinoriChang, Chien-ChungNishimoto, YoshioChang, Ching-yaoNishiwaki, HisashiChang, Dong WookNishiyama, YusukeChang, Dong-JoNisnevitch, MarinaChang, Fang RongNissan, JosephChang, Geng-RueiNistor, CristinaChang, Hai-ChouNitulescu, George MihaiChang, Hao-XunNo, Joo HwanChang, Hsueh-WeiNobili, StefaniaChang, Kee-LungNobre, BeatrizChang, MinsunNoda, HidetoshiChang, Sue-JoanNogales-Bueno, JulioChang, Te-ShengNoguchi, Constance TomChao, Chih-HuaNorman, TrevorChao, Louis KuopingNormand, BernardChao, Pi-YuNorouzitallab, ParisaChapman, EliNorval, MaryCharline, KiefferNouranian, SasanChartier, AgnesNovak, LajosChase, Mark W.Novikov, AlexanderChass, GregoryNovikova, Nataliya E.Chatterjee, JhinukNovinec, MarkoChatterjee, RuchiraNovio, FernandoChatterjee, SumanNovío, SilviaChatterley, Adam SNowacka-Jechalke, NataliaChau, Chi-FaiNowak, AdrianaChaung, Hso-ChiNowak, RenataChaves, SílviaNowicka, AnnaChaves-Lopez, ClemenciaNowicka, GrażynaChavez, FermanNowicki, JanuszChazot, Paul LNtie-Kang, FideleChe, C.-T.Ntougias, SpyridonChe, LixiaoNüesch, FrankCheca, AntonioNuhn, LutzChecchetto, RiccardoNuić, IvonaChelbi, Sonia T.Núñez, OscarChemat, FaridNurieva, EvgeniyaChen, ChiachungNürnberg, DennisChen, Chun-ChiNycz, Jacek EChen, Chung-YiO’Duill, Miriam L.Chen, Chun-JungObata, YasukoChen, GuangpingObrador, ElenaChen, GuoxunOda, YukariChen, HaixiaOdenthal, MargareteChen, HaoyuanO'Doherty, GeorgeChen, HongweiOfford, JamesChen, Hsin-ChunOfir, RivkaChen, Hsing-YinOgasawara, HirohitoChen, HuaiqiongOgasawara, ToruChen, HuixiongOgawa, AkiyaChen, Jen-TsungOgino, ToshioChen, Jhy-DerOgnyanov, ManolChen, JiahaoOgórek, RafałChen, Jiann-ChuOgungbe, IfedayoChen, JianzhongOgunjirin, AdebowaleChen, Jing-HsienOh, Kyung TaekChen, JunOh, KyungsooChen, Kew-YuOh, Man-hoChen, Kun-MingOh, SeikwanChen, Kuo-YuOh, Seung JaeChen, Liang-YuO'Hagan, DavidChen, Lih-GeengOhnishi, MasatoshiChen, LinOhta, ShinjiChen, MingOhta, YoshihiroChen, MingshengOjala, JohannaChen, Min-HueyOjeda-May, PedroChen, MohanOjika, MakotoChen, Po-YuanOjima, KoichiChen, QiOka, NatsuhisaChen, Qiao-HongOkada, TaketoChen, Ruei-mingOkawara, ToruChen, Shih-YuOkazawa, AtsushiChen, ShiyuO'Keefe, BarryChen, Shuo-binOkino, TatsufumiChen, WenchunOkovytyy, SergiyChen, XiOkuda, MasayukiChen, XiaoyanOkumura, NobuakiChen, Yu-ChihOkunade, Adewole L.Chen, Yu-KuoOlaru, Octavian TudorelChen, Yung-ChihOlatoye, Marcus O.Chen, Yun-WenOlech, MartaChen, ZhaoOlejar, Kenneth J.Chen, ZheOlejniczak, IwonaChen, ZhihangOlejnik, AnnaChen, ZhijieOlejnik, MalgorzataChen, ZhonghuaOleskin, Alexander V.Chen, ZhongningOliva, LeoneChénais, BenoitOliva, RominaCheng, Bing-MingOliveira, BeatrizCheng, Chih-ChiaOliveira, Hugo M.Cheng, GangOliveira, JoanaCheng, Heung-ChinOliveira, PedroCheng, Hung-ChiOliveira, RodrigoCheng, Juei-TangOliveira, RuiCheng, Jya-WeiOliveira-Santos, Sonia AndreiaCheng, PeiOliveri, PaoloCheng, QingqingOliverio, ManuelaCheng, Qing-QingOliver-Ortega, HelenaCheng, Sen-SungOliviero, GiorgiaCheng, ShouyunOlson, KennethCheng, Wei-chiehOmar, Syed HarisCheng, XingguoOmbra, Maria NeveCheng, ZhihongOms, OlivierChengwen, SunOnchoke, KefaCheon, Choong-illOnduka, ToshimitsuCheong, Yong HwaO'Neil, GregoryCheong, Yuen KiOniga, IlioaraCherkaoui-Malki, MustaphaOniszczuk, AnnaChermette, HenryOnnis, ValentinaChern, Ming-KaiOnorato, Amber J.Chetwynd, AndrewOnuta, Marie-ChristineChi, Celestine N.Onysko, PetroChia, Win-LongOon, Hazel H.Chiang Hsieh, Lin-HanOpara, Elizabeth I.Chiang, Anthony S.T.Operamolla, AlessandraChiang, Hsiu-MeiOracz, JoannaChiang, Long Y.O'Reilly, ElaineChiang, Meng-TsanOri, OttorinoChiang, Ming-HsiOrian, LauraChiang, Tai-anOrlando, TomasChiang, Yi-TingOroian, MirceaChien, Huan-ChiehOroz-Guinea, IsabelChien, Shih-ChangOrsi, MarioChiessi, EsterOrte, AngelChin, Young-WonOrtega, Angel L.Chinchilla, RafaelOrthaber, AndreasChiodo, FabrizioOrtiz, Maria J.Chiou, Chun-TangOsborn, HelenChirumbolo, SalvatoreOsborne, JamesChisholm, John D.Ośmiałowski, BorysChitchumroonchokchai, ChureepornOsolodkin, DmitryChiu, Chi-chengOsório, Wislei RChiu, Tai-chiaOstacolo, CarmineChiu, Tsai-HsinOstaszewski, RyszardChiulan, IoanaOstovar, SaeidehChiva-Blanch, GemmaOstrowska, KingaChizzola, RemigiusOstrowski, StanisławChladek, GrzegorzOtero, CarolinaChlebek, JakubOtero, MartaChlichlia, KaterinaOtmačić Ćurković, HelenaChmiel, TomaszOtsuka, MasamiChmielarz, PawełOtsuka, YuzuruChmielewska, AleksandraOtt, MariaChmielewska, EwaOu, Tian-miaoChmurzyńska, AgataOwczarek, AleksandraCho, Cheong-WeonOwen, JeremyCho, Dae WonOwusu-Apenten, RichardCho, EunaeOyama, DaiCho, Hea-YoungOzaki, ToshinoriCho, MichaelOzawa, ShogoCho, Nam-JoonOzen, Mehmet OzgunCho, Seung-SikOzkan, Sveta ZhiraslanovnaCho, SungeunPace, VittorioCho, SunghunPachapur, VinayakCho, SungyunPacheco, Fabio JulianoChobot, VladimirPacheco, NeithChoi, Hyo-JickPachuta-Stec, AnnaChoi, Jae SuePaci, MaurizioChoi, Jae-HongPacureanu, LilianaChoi, Ki ChoonPadmanabhan, JagannathChoi, Min-KooPadmanabhan, ParasuramanChoi, Young HaePaduano, AntonelloChoi, Young HeePaduch, RomanChoi, Yung HyunPaeng, Ki-JungChojnacka, AnnaPagani, StefaniaCholewinski, GrzegorzPage, MelissaChollet, Jean-FrançoisPage, Richard C.Chong, ParksonPaggetti, JérômeChoquesillo-Lazarte, DuanePagnotta, Mario A.Chou, Shih-FengPaixao Coelho, Jose AugustoChou, Y.C.Pakulski, ZbigniewChoudhary, VivekPal, RumpaChow, Aviva Shing FungPal, SudiptoChow, Simon Kwoon-HoPalacios, OscarChrissopoulou, KiriakiPalacios, SaraChriste, Karl O.Palage, MarianaChristensen, Jørn B.Pałasz, ArturChristensen, LarsPalenzuela López, José AntonioChristians, JulianPalermo, EdmundChristians, UwePalinko, IstvanChristodoulou, Maria-IoannaPalla, FrancoChristoforides, EliasPallaoro, AlessiaChristopoulos, M. V.Paller, ChanningChristova-Bagdassarian, ValentinaPalma, MiguelChrobak, ElwiraPalma, VincenzoChrobok, AnnaPalmer, AndrewChruszcz, MaksymilianPalomo, JoseChu, JiaxiangPalomo, ValleChu, ShidongPalumbo, CarlaChu, Yen-WeiPampana, SilviaChua, BeeleePan, GuoqingChuck-Hernández, CristinaPan, LinqiangChuffa, Luiz GustavoPan, XiaoyongChulhai, Dhabih VPan, Y. J.Chun, Heung JaePan, YangangChung, Byung MinPanagiotidis, MihalisChung, Hyun-JungPanaro, Maria AntoniettaChung, Jing-GungPanay Escobar, Aram JoelChung, Ying-ChienPanchal, Sunil KChunsuo, YaoPanderi, IreneChuyen, Hoang V.Pandey, Ramesh PrasadChyau, Charng-CherngPaneth, AgataCiaccheri, LeonardoPaneth, PiotrCiancaleoni, GianlucaPanfoli, IsabellaCianciosi, DanilaPanniello, AnnamariaCiccone, Marco MatteoPannkuk, Evan L.Çiçek, Serhat SezaiPantani, RobertoCicero, ArrigoPantos, DanCicero, Arrigo Francesco GiuseppePanzella, LuciaCichero, ElenaPaoli, PaolaCichorek, MiroslawaPaoli, PaoloCiecholewski, MarcinPaolillo, MayraCieniewski-Bernard, CarolinePaolo Busardó, FrancescoCieplik, FabianPaolone, AnnalisaCiereszko, IwonaPapadimitriou, KonstantinosCież, DariuszPapageorgis, PanagiotisCigáň, MarekPapaioannou, DionissiosCihacek, LarryPapakyriakou, AthanasiosCilla, AntonioPapamichael, EmmanuelCimmino, GiovanniPapetti, AdeleCimpoiu, ClaudiaPapini, EmanueleCinelli, PatriziaPapo, NivCinteza, Ludmila OtiliaPapoutsis, KonstantinosCiobica, AlinPapp, AndrásCiofani, GianniPappa, AglaiaCiofi-Baffoni, SimonePappas, ChristosCione, ErikaPáramo-García, UlisesCirri, DamianoParashar, DeepakCirrincione, GirolamoParaskevopoulou, PatrinaCisowsky, JaroslawParcells, MarkCitterio, BarbaraParedes, José ManuelCiulu, MarcoParikesit, Arli AdityaČizmić, MirtaParisini, EmilioClaridge, Jolyon KPark, Chung GyooClark, AndrewPark, Dae-HunClark, TimothyPark, Dong SunClarke, RonPark, EnochClaus, HaraldPark, Hyung-HoClausen, Rasmus PrætoriusPark, Il-KwonClement, CristinaPark, Jeong HoClericuzio, MarcoPark, JiyoungClose, David M.Park, Jong-InCobo, MarthaPark, Jong-SeungCoburn, JeanninePark, JoshuaCoccheri, SergioPark, Ky YoungCoccia, GianlucaPark, Kyoung ChanCocetta, GiacomoPark, Min-JungCochis, AndreaPark, Myung HwanCochran, BlakePark, SimonCochrane, StephenPark, WansuCodacci-Pisanelli, GiovanniPark, YongsoonCodee, JeroenParker, Stewart F.Coelho, CarolinaParlea, LorenaCoen, De GraafParolini, CinziaCoey, J.M.D.Parra, AlejandroColdea, TeodoraParra, RobertoCole, Kathryn E.Parrella, EdoardoColecchia, DavidParrilli, AnnapaolaColl, JosepPârvu, Alina ElenaCollier, JasonPascal, SimonCollina, SimonaPascali, GiancarloCollins, Stuart G.Pascarelli, Nicola AntonioCologna, StephaniePáscoa, RicardoColombo, RaffaellaPaseiro-Cerrato, RafaelColonna, GiovanniPasick, JohnColquhoun, Thomas A.Pasini, DarioColuccia, MauroPasini, LuigiComai, StefanoPasqua, GabriellaComito, Robert JPasquale, Claudio DeCommisso, MauroPasqualone, AntonellaCompostella, FedericaPassamonti, SabinaConceição, KatiaPassos, FabianaConcepción Heydorn, PatriciaPastore, AnnalisaConcilio, SimonaPaszkiewicz, SandraConnolly, JosephPátek, MiroslavConstantin, LucianPatel, AlokConstantinescu, Stefan N.Patel, AnamikaConstantinou-Kokotou, ViolettaPatel, BhargavConte, GiuseppePatel, HardikkumarConte, LanfrancoPatel, Yashomati M.Conti, BarbaraPaterson, BrettContiero, JonasPathakoti, KavithaContino, MarialessandraPathi, SumanCopolovici, Dana MariaPatil, Avinash J.Corato, Riccardo DiPatra, BarunavaCorazza, Marcos LúcioPatra, Jayanta KumarCorazzin, MircoPatrauchan, Marianna A.Corbett, CharlottePatrizia, NittiCordaro, MarikaPattabiraman, MaheshCordeiro, Rodrigo MaghdissianPatterson, Adam V.Corke, HaroldPatyra, EwelinaCornejo, JavieraPauer, WernerCoronas Ceresuela, JoaquínPaul, AnanyaCorradini, DaniloPauliukaite, RasaCorradini, RobertoPau-Roblot, CorinneCorradini, ValdisPavela, RomanCorrales-Garcia, JoelPavic, ValentinaCorrea, ArkaitzPawelkowicz, MagdalenaCorreia, Ana CristinaPawlicki, MiłoszCorreia, IlidioPayne, AnnetteCorrigan, DamionPayrastre, BernardCorsaro, CarmeloPazdro, RobertCortés Castell, ErnestoPazourek, JiriCosco, DonatoPeana, Massimiliano FCosta Lima, SofiaPeczuh, MarkCosta Moutinho, FabíolaPedatella, SilvanaCosta, Blaise MathiasPedersen, AndersCosta, GeisonPedraza-Chaverri, JoseCosta, Maria-margarida R. R.Pedretti, AlessandroCosta, Paulo JorgePedrosa, Mercedes M.Costa, PedroPedrosa, RafaelCosta, RosariaPedroza, MesiasCosta, StefaniaPeeters, MarloesCostantino, LucaPeifer, ChristianCouce, María D.Peigneur, SteveCouderc, FrançoisPelagalli, AlessandraCoulon, StéphanePelegov, DmitryCoutinho, Paulo José GomesPeleteiro, MercedesCowen, Bryan J.Pelin, MarcoCoy-Barrera, EricssonPellegrino, SaraCoyne, MarkPellei, MauraCozzolino, AnthonyPellis, AlessandroCozzolino, DanielPelosi, ClaudiaCragg, PeterPeluso, IlariaCrascì, LuciaPena-Pereira, FranciscoCravero, Maria CarlaPeña-Ruiz, TomásCrawford, Philip W.Pence, BrandtCreamer, RebeccaPenczek, StanislawCrestini, ClaudiaPendleton, PhillipCreus, MarcPeng, JunCrișan, GianinaPeng, MengfeiCrisan, OvidiuPeng, Shu-FenCrnkovic, AnaPeng, TengCroce, Anna CletaPeng, ZhiliCrocket, KirstyPenke, BotondCroset, MartinePentak, DanutaCrowley, JamesPentzold, StefanCrowley, PeterPepeu, GiancarloCrucho, CarinaPepi, MilvaCrucho, Carina Isabel CorreiaPeppel, TimCruz, AdrianoPeppelenbosch, MaikelCruz-Sosa, F.Perche, FédéricoCsámpai, AntalPerdih, AndrejCuadrado, AngelesPerego, CarlaCucciniello, RaffaelePereira, DavidCueto, MercedesPereira, EduardoCui, TiantianPereira, Eliseu J.G.Cui, XinPereira, FlorbelaCui, YiPereira, Frederico C.Cui, ZhibinPereira, J.A.Cullen, CarliePereira, José AugustoCulot, MaximePereira, Maria Do CarmoCunha, Luís MiguelPereira, Olívia R.Cupellini, LorenzoPereira, VandaCurti, ClaudioPerestrelo, RosaCurti, DanielaPérez Sestelo, JoséCustódio, DaniloPérez, María J.Cutrignelli, AnnalisaPerez, SergeCycoń, MariuszPérez-Álvarez, Jose AngelCzaplewski, CezaryPerez-Jimenez, JaraCzarnocki, ZbigniewPerez-Stable, CarlosCzechowski, TomaszPérez-Villanueva, JaimeCzekelius, ConstantinPeri, FrancescoCzerniewicz, PawełPerić, MihaelaCzerwinska, MonikaPerinelli, Diego RomanoCzjzek, MirjamPerlepes, SpyrosCzopka, TimPermyakov, EugeneCzuba, ZenonPerna, FilippoCzyżowska, AgataPerrakis, AnastassisD’ Alessandro, AngeloPerreault, JonathanD’Anna, FrancescaPerteghella, SaraDa Motta, M.Pertusati, FabrizioDa Silva, Mateus WebbaPeruzzi, FrancescaDa Silva, Monique Gabriella AngeloPešić, MilicaD'Abrosca, BrigidaPestov, AlexandrD'accolti, LuciaPeter, Emanuel K.Dacheux, Jean-LouisPeter, KatherineDachineni, RakeshPETER, Martin G.Dagorne, SamuelPeterson, JuliaD'Agostino, NunzioPeterson, LarrynDai, Yang DPetit, Patrice X.Dai, YuminPetkova, NadezhdaDai, ZhigaoPetrella, AndreaDal Ben, MatteoPetriccione, MilenaDal Piaz, FabrizioPetrikaite, VilmaDallacosta, LorenzaPetrillo, GiovanniDall'Acqua, StefanoPetropoulos, SpyridonDall'Asta, ChiaraPetrov, DmitryDallavalle, SabrinaPetrova, Krasimira T.Dallemagne, PatrickPetruczynik, AnnaDalmoro, AnnalisaPetruk, GannaDalpozzo, RenatoPeyton, David H.Daly, NorellePezzolla, DanielaDamalanka, VishnuPezzotti, GiuseppeDamiani, DanielaPezzuto, AldoDamjanović, MarkoPfeffer, GeraldDamjanovski, SashkoPhilips, NeenaDamkaci, FehmiPhuoc, Nghia TruongDa'na, EnshirahPiao, XijunDanciu, CorinaPiastowska-Ciesielska, Agnieszka WandaDandekar, ThomasPiccialli, VincenzoDanezis, GeorgiosPiccionello, Antonio PalumboDang, Jing-ShuangPiccirillo, ClaraDangles, OlivierPickova, JanaDanielak, DorotaPicmanova, MartinaDaniele, AuroraPicone, GianfrancoDaniellou, RichardPidatala, Venkata RamanaDanielson, NeilPielichowski, KrzysztofDaniels-Wells, Tracy R.Piemontese, LucaDansette, Patrick M.Pieraccini, StefanoDante, SilviaPiergiovanni, LucianoDarafsheh, ArashPierpaoli, ElisaD'Argenio, ValeriaPierre, GuillaumeDarvesh, SultanPietrella, DonatellaDas, AninditaPietrzyk, BozenaDasgupta, KasturiPigge, F. ChristopherDasgupta, SantanuPigłowski, MarcinDasgupta, SubhamPignitter, MarcDash, Alekha K.Pihlava, Juha-MattiDash, BirajaPilkington, LisaDash, RanjeetPilo-Pais, MauricioDatta, KausikPINA, FernandoDaugrois, Jean-HeinrichPina, JoãoD'Auria, EnzaPincus, SethDavari Esfahani, M. MahdiPindi, SureshDavid-Cordonnier, Marie-hélènePineda, AntonioDavies, ChristopherPineda, MiguelDavinelli, SergioPiñeiro, MartaDavis, Keith R.Pinela, JoséDavis, MarkPingault, LiseDawid, CorinnaPinheiro, MarinaDawn, ArnabPinnapireddy, Shashank ReddyDe Almeida Coelho De Sousa, Maria JoãoPino, Manuel M. Sánchez DelDe Almeida, AndreiaPintea, AdelaDe Alvarenga, Elson SantiagoPinto Carvalho, Ana MariaDe Amici, MarcoPinto Reis, Catarina PintoDe Andrade, Sara Adrián LópezPinto, Diana C. G. A.De Aza, Piedad N.Pinto, MafaldaDe Barros Fernandes, PedroPinto, MilenaDe Bellis, LuigiPinto, PaulaDe Berardis, DomenicoPinto, UelintonDe Bock, MarijkePinu, Farhana R.De Caro, VivianaPinzaru, IuliaDe Carvalho Santos Ebinuma, ValériaPiotrowska, UrszulaDe Castro, SoniaPiotrowski, PiotrDe Deurwaerdère, PhilippePiovesana, SusyDe Diego, MartaPires, Regina HelenaDe Feo, VincenzoPirillo, AngelaDe Giudici, GiovanniPiringer, MartinDe Gonzalo, GonzaloPiriou, YannickDe Jesus, ErnestoPiro, GabriellaDe Jonghe, StevenPisano, Pablo L.De Kimpe, NorbertPisarska, JoannaDe La Garza, MireyaPisklak, Dariusz MaciejDe La Lande, AurélienPissarek, MargitDe La Torre, Maria Del CarmenPitto, LetiziaDe Lacey, Antonio LopezPitucha, MonikaDe Lago, EvaPiva, TerrenceDe Leo, MarinellaPivetta, TizianaDe Marco, RossellaPivonello, RosarioDe Masi, LuigiPizzi, AntonioDe Matteis, ValeriaPizzorno, Mario AndresDe Mendonça, Dina I. M. D.Plácido-Escobar, Jersson E.De Mieri, MariaPla̧der, WojciechDe Oliveira Carvalho, Carla RobertaPlanas, MartaDe Oliveira, KleberPlano, DanielDe Paula Venancio, ViniciusPlastina, PierluigiDe Rosa, Maria CristinaPlate, ManuelaDe Simeis, DavidePlatts, James A.De Sousa, Frederico B.Plavec, JanezDe Sousa, Maria EmiliaPlaza, AlbertoDe Tullio, M.C.Plażuk, DamianDe Vendittis, EmmanuelePlessas, StavrosDe Visser, SamuelPlíhalová, LucieDe Vleeschouwer, FreijaPlonka, PrzemyslawDe Vries, CarliePłotka-Wasylka, JustynaDe Wit, MarynaPlotnikov, EgorDe With, GijsbertusPluquet, OlivierDealy, Caroline N.Plutino, Maria RosariaDean, LisaPlyusnin, Victor F.Dean, Lisa OehrlPoater, AlbertDeb, SubrataPoczai, PéterDebnath, Asim KumarPoddel'sky, AndreyDecristoforo, ClemensPogorzelska, AnetaDeDiego, Marta L.Pogorzelska-Nowicka, EwelinaDegano, IlariaPohl, PawelDegennaro, LeonardoPoinsot, VerenaDegiacomi, Matteo T.Poitras, DanielDegola, FrancescaPoku, Rosemary A.Dehaen, WimPolakova, MonikaDehzangi, AbdollahPolanski, MarekDeineka, VictorPolavarapu, LakshminarayanaDejam, MortezaPolavarapu, Prasad L.Del Giudice, RitaPoletto, MatheusDel Río Celestino, MercedesPoli, GiulioDel Rio, DanielePoliak, PeterDelannoy, EtiennePolidori, M. Cristina Delannoy, PhilippePolischuk, PavelDelaude, LionelPolishchuk, P.Delbecq, FrédéricPoliteo, OliveraDelfin, Dawn A.Pollmann, KatrinDella Ca', NicolaPollock, Richard L.Delogu, GiovannaPolo, Beatriz De Las HerasDelorme, VincentPolyak, StevenDelville, Marie-HélènePolyakov, NikolayDembska, Anna RenataPoma, PaolaDemchenko, Alexei V.Pomerantz, WilliamDemir, FatihPomin, Vitor H.Demonacos, ConstantinosPons, JosefinaDemopoulos, Vassilis J.Pons, MiquelDen, WalterPooler, DarcyDenat, FranckPoór, MiklósDeng, JianjunPoozesh, SadeghDeng, JingPopa, Mona ElenaDeng, QingPopiołek, ŁukaszDeng, RuixiaPoplawski, TomaszDeng, XiaoranPopović-Djordjević, Jelena BDenis, SerenoPopovich, DavidDeo, PermalPorcaro, FrancescoDeokar, HemantkumarPorchia, MarinaDepeint, FlorePorciani, DavidDeplazes, EvelynePorebski, GrzegorzDeplus, RachelPortaro, FernandaDeRosa, ChristopherPorto, André Luiz MeleiroDesai, JigarPotapov, AndreiDesai, Umesh R.Potparević, BiljanaDesando, GiovannaPotterat, OlivierDeshmukh, RupeshPoudel, Tej NarayanDeshpande, RahulPouli, NicoleDeshpande, ShayuPountney, DeanDessolin, JeanPoupot, RemyD'Este, MatteoPourcet, BenoitDetsi, AnastasiaPourquier, PhilippeDeuss, PeterPourrahimi, Amir MasoudDevadas, KrishnakumarPowell, MichaelDevasurendra, AmilaPozo, Óscar J.Dey, GangotriPrajzler, VaclavDeYonker, Nathan J.Pranovich, AndreyDhakal, DipeshPrassl, RuthDhakshinamoorthy, AmarajothiPratsinis, HarrisDhanasekaran, Danny N.Preite, MarceloD'hulst, ChristophePrenzler, PaulDi Bella, SantoPrescott, John F.Di Biase, StefanoPreti, DeliaDi Buduo, ChristianPriel, AviDi Bussolo, ValeriaPrieto, Maria PilarDi Carlo, MartaPrieto-Lloret, JesusDi Donato, MarziaPrim, DamienDi Gioia, Maria LuisaPrima, Giulia DiDi Liegro, ItaliaPrimožič, InesDi Luca, MariagraziaPrinsi, BhaktiDi Luccia, AldoPrior, SarahDi Marino, DanielePrisic, SladjanaDi Mauro, Maria DomenicaProchazka, DavidDi Natale, ConcettaProestos, CharalamposDi Pasqua, Anthony J.Prohens, JaimeDi Pierro, ProsperoProietti, SilviaDi Sotto, AntonellaProkai, LaszloDi Stefano, AntonioProsen, HelenaDi Tommaso, DevisProserpio, DavideDiaba, FaïzaProtti, MicheleDiamond, GillPrudent, MichelDiana Camelia, NuţǎPruess, Birgit M.Diana, RositaPruvost, AlainDias, Rolando CSPrzybylski, PiotrDíaz, CristinaPrzybyt, MałgorzataDiaz, GasparPsurski, MateuszDíaz, JoséPu, JingzhiDichiara, AnthonyPucheault, MathieuDickert, Franz L.Puglia, DeboraDiculescu, Victor ConstantinPuglisi, RitaDie, JosePuglisi-Weening, MelanyDiekman, AlanPuiggalí, J.Díez, DavidPuiggalí, JordiDijkhuizen, LubbertPuizina, JasnaDijkstra, Bauke W.Punganuru, Surendra ReddyDilonardo, ElenaPurchas, Roger W.Dimakis, NicholasPuttreddy, RakeshDimitrakopoulos, GeorgiosPutz, Mihai V.Dimitroff, Charles J.Py, SandrineDina, Nicoleta ElenaPyka-Pająk, AlinaDinadayalane, TandabanyPytka, KarolinaDing, BaoqingPyzik, MichalDing, ChunbangQaim, Syed M.Ding, JianxunQian, PengxuDing, LingQiao, LiangDing, Wei DanQin, SanboDing, ZhaoyangQiu, HuanDinica, Rodica MihaelaQuadrelli, PaoloDinis, Maria Alzira PimentaQuan, YangjianDiniz, CarmenQuartu, MarinaDiniz, Paulo Henrique Gonçalves DiasQueiroz, Maria-JoaoDinu-Pîrvu, Cristina ElenaQuesada Pérez, Víctor ManuelDionisio, GiuseppeQuiles, José L.Diretto, GianfrancoQuillard, ThibautDirsch, VerenaQuinn, Mark T.Disdier, ClemenceQuintard, Jean-PaulDixit, MuditQvortrup, KatrineDixon, DavidR. Odell, LukeDixon, David A.R. Santos, Cleydson BrenoDmitrenok, Pavel S.Raabe, GerhardDo Carmo, Sergio J. C.Rabanal Anglada, FrancescDobrovolskaia, MarinaRabbani, GulamDobrzynski, PiotrRace, PaulDoda, Sai ReddyRachek, LyudmilaDoddapaneni, RaviRachwalski, MichałDodero, VeronicaRácz, AnitaDoerksen, John RobertRadchenko, Eugene V.Döhler, DianaRadhakrishnan, SivaprakasamDoi, HisashiRádis-Baptista, GandhiDoi, TakayukiRadmila, PavlovicDolashka, PavlinaRadošević, KristinaDoležal, KarelRadoshevich, LillianaDolga, AmaliaRadu, Gabriel-LucianDolzhenko, AntonRadwan, MohamedDomine, MarceloRagauskas, Arthur J.Domínguez-Álvarez, EnriqueRagg, EnzioDominikowska, JustynaRagno, GaetanoDonahue, MatthewRagno, RinoDonato, RosarioRagupathy, ViswanathDong, ShihuiRagusa, AndreaDong, YiboRagusa, Maria AntoniettaDong, YueshengRahimi, FaridDonnarumma, GiovannaRahman, Khondaker MirazDonno, DarioRahman, Khondokar MizanurDoolittle, Russell FRahman, Masmudur M.Doran, ToddRahman, ZiaurDoria, FilippoRahmanPour, RahmanDoriano Bianco, ArmandoRahme, KamilDorin, JuliaRai, DilipDorotkiewicz-Jach, AgataRai, PrakashDos Santos Niculau, EdenilsonRaimondi, DanieleDos Santos, DemetrioRaina, MedhaDos Santos, José C.S.Raines, RonaldDos Santos, Reinaldo SousaRaingeaud, JoëlDoseff, AndreaRajauria, GauravDosoky, Noura S.Rajca, MariolaDossena, SilviaRajčević, NemanjaDouris, VassilisRaje, MithunDovinova, ImaRajnarayanan, RajendramDoyle, SiamsaRakitin, Oleg A.Dračínský, MartinRakotondraibe, Harinantenaina LivaDragomir, MihneaRalston, EmilyDrasar, PavelRamachandran, AnupDrewniak, SabinaRamaiahgari, Sreenivasa C.Drozdov, Aleksey D.Ramakrishnan, SrinivasanDrummond, RebeccaRamalho, TeodoricoDu, DeguoRamanavicius, ArunasDu, FanfanRamaraj, PanduranganDu, GuodongRamasamy, ElamparuthiDu, Shu ShanRambaldi, MirellaDu, YongleRamesh, SreerangappaDuarte Campos, Daniela FilipaRamil, CarloDuarte, AndreiaRamirez-Vick, JaimeDuarte, Maria CristinaRamis, GianguidoDuarte-Carvajalino, Julio M.Ramona, PaltineanDubey, RaminRamos, PedroDubielecka, Patrycja M.Rana, DipakDudas, JozsefRanc, VáclavDudnikov, Alexander Ju.Rangel-Hernandez, VictorDueñas-Patón, MontserratRangnekar, AbhijitDüfer, MartinaRanzato, EliaDufossé, LaurentRao, VidhyaDuggan, Brendan M.Rapeanu, GabrielaDuh, Pin-DerRaposo, Maria Filomena De JesusDuma, LuminitaRapposelli, SimonaDumas, FrançoiseRaptopoulos, GrigoriosDumur, FrédéricRascio, AgataDunckley, TravisRascon, AgustinDundas, IanRastija, VesnaDuong, HaiminhRatajewski, MarcinDuong-Quy, SyRateb, Mostafa E.Dupont, LaurentRathinasabapathy, AnandharajanDurães, LuisaRatkova, Ekaterina L.Durand, Jean-OlivierRatnayake, RanjalaDurán-Peña, María JesúsRaudino, AntonioDurazzo, AlessandraRaugei, GiovanniDurek, ThomasRaupp, Sebastian M.Durgo, KsenijaRauwel, ProtimaDutartre, PatrickRavegnini, GloriaDutoi, Anthony D.Ravelli, DavideDutta, ArijitRavenscroft, NeilDutta, BiswanathRaviola, CarlottaDutta, DebarunRawel, HarshadraiDutta, PalashRayan, AnwarDuval, Raphaël E.Rayson, Gary D.Duvall, MelvinReader, Jocelyn C.Dvir, HayRebelo, SusanaDvoyashkin, MuslimRebl, AlexanderDwivedi, ChandraharReboredo-Rodríguez, PatriciaDworak, AndrzejReboud, JulienDyguda-Kazimierowicz, EdytaRebros, MartinDzidic, AlenRecabarren Gajardo, GonzaloDziedzic, ArkadiuszReddy, SakamuriDziegielewska, BarbaraReetz, Manfred T.Dziewit, LukaszReeve, VivienneDziki, DariuszRegal, PatriciaDzikovski, BorisReginato, Mauricio J.Dzimitrowicz, AnnaReguero, MarDziuba, DmytroRehrig, Erin M.Dzolic, ZoranReich, RobertDzuba, Sergei AndreevichReichert, David E.Ebani, VelentinaReid, Christopher W.Ebrahim, WeaamReina, Tomas RamirezEbrahimi, AidaReinaud, OliviaEchevarria, AureaReis, CelsoEckl, PeterReiss, Guido J.Edafiogho, IvanReissmann, SiegmundEdalati, KavehReiter, RusselEder, Iris E.Remaut, KatrienEder, MatthiasRemesar, XavierEdin, Nina JeppesenRemize, FabienneEdlund, UlricaRen, YulinEdmondo, BattistaRenata, HansEdreira, Martin M.Renault, NicolasEdström, DanielRenčiuk, DanielEdwards, MattRendle, Phillip M.Efimov, AlexanderRenman, GunnoEfremenko, ElenaRenner, UlrichEgami, HiromichiRenzi, PolyssenaEgerszegi, IstvánRescifina, AntonioEggeling, LotharReshkin, Stephan J.Egodawatte, ShaniRevilla, EugenioEguiraun, HarkaitzRey, AlejandroEhlers, PeterRey, LuisEhlert, ClaudiaReyes, FernandoEhm, ChristianReyes-Chilpa, RicardoEhrich, MarionReyes-Garcés, NathalyEhrlich, GarthReynisson, JóhannesEid, NabilReynolds, AndrewEiroa, José LuisRey-Stolle, FernandaEisenreich, WolfgangRezaei-Ghaleh, NasrollahEjfler, JolantaReznik, SandraEjidike, Ikechukwu P.Rezzani, RitaEkielski, AdamRhee, Hyun-WooEkiert, HalinaRhee, Jin-KyuEklund, Patrik C.Rho, Hoon SukElaissari, AbdelhamidRho, Jung-RaeEl-Bacha, TatianaRhodes, Diana C. J.Eldeeb, MohamedRibeiro, ClaudiaEleftheriou, Eleftherios P.Ribeiro, JoãoElemans, J.A.A.W.Rice, JohnElghobashi-Meinhardt, NadiaRicelli, AlessandraElkordy, AmalRicha, TambiEller, FredRichard, JohnElleuche, SkanderRichard, MandleElli, StefanoRichardson, AlanElmann, AnatRichomme, PascalEl-Mashtoly, Samir F.Richter, KatrinEl-Safty, Sherif A.Richter, ShacharEl-Seedi, Hesham R.Riedl, RainerElshafie, Hazem S.Rienzo, MonicaElshahawi, SherifRigamonti, LucaElsinghorst, Paul W.Rigano, DanielaEmerson, JosephRigano, Maria ManuelaEmery, NeilRigo, EvelineEmpl, MichaelRijo, PatríciaEnchev, VenelinRimondini, LiaEndo, TakeshiRinnerthaler, MarkEndres, KristinaRíos-Gutiérrez, MarEngel, MatthiasRishi, ArunEngel, Paul C.Risoluti, RobertaEngle, JonathanRita Costa-Pinto, AnaEnguita, Francisco J.Ritala, MikkoEnkvist, ErkiRitch, JamieEnnas, GuidoRitzoulis, ChristosErdem, EmreRivas, FatimaErsan, SevcanRivera De La Rosa, JavierEsadze, AlexandreRivera-Monroy, Zuly JennyEscalante, CarlosRivero, SoniaEscalante, JaimeRizvi, SyedEscames, GermaineRizzo, AntonioEscario, JoséRizzolio, FlavioEscorihuela, JorgeRoach, ThomasEslami, HosseinRobbennolt, ShaunaEspada, JesúsRobert, EricEspinosa-Diez, CristinaRoberts, Sue A.Esposito, Emilio XavierRobertson, AvrilEspuelas, SocorroRobertson, Mark J.Esque, JérémyRobiette, RaphaelEsquinazi, PabloRobinson, SaraEstelrich, JoanRobledo, Virginia RodríguezEsteve-Romero, JosepRobles Medina, AlfonsoEsteves, Ana CristinaRobles, EduardoEstévez Cabanas, Ramón J.Robson, MatthewEtacheri, VinodkumarRocca, CarmineEu, PeterRoccatano, DaniloEvans, Joseph L.Rocchetti, GabrieleEves-van Den Akker, SebastianRocchigiani, LucaEvtugyn, GennadyRocha, Hugo A. O.Ezekiel, UthayashankerRocha-Martin, JavierEzquerra-Brauer, Josafat-MarinaRochefort, SimoneF. Rasulev, BakhtiyorRocio-Bautista, PriscillaFabiani, RobertoRoderburg, ChristophFabiano, EduardoRodrigues, António S.Facão, JorgeRodrigues, FranciscaFaggio, CaterinaRodrigues, JoanaFailla, Cristina M.Rodrigues, JoaoFairlamb, AlanRodrigues, OlivierFaldyna, MartinRodríguez García, Mario EnriqueFalentin-Daudré, CélineRodriguez Lorenzo, Luis MariaFales, AndrewRodriguez, Hortensia M.Fallon, ThomasRodriguez, Juan B.Falqué, ElenaRodriguez, NoelFan, ChenguangRodríguez, Teresa PinedaFan, WeizhengRodriguez-Cueto, CarmenFan, XiulinRodríguez-Gómez, ArturoFan, YunchangRodríguez-Pérez, CeliaFan, ZhijinRodríguez-Pérez, José ManuelFanali, ChiaraRodríguez-Roque, María JanethFanfrlík, JindřichRodziewicz, PawelFang, MarongRoelen, BernardFang, MingxiRoglans, AnnaFang, XiRogozhin, EugeneFang, XiaolanRoh, ChanghyunFang, YiRoh, JaroslavFanizzi, Francesco PaoloRoh, SanghoFantacuzzi, MarialuigiaRohs, RemoFarag, MohamedRoilo, DavidFarantos, Stavros C.Roj, EdwardFarcas, Anca CorinaRollin, PatrickFardel, OlivierRollini, ManuelaFare, SilviaRomán Guerrero, AngélicaFarhad, AliRomanelli, Maria NovellaFarhat, GraceRomano, NiclaFaria, AnaRomanov, Georgy A.Farjon, JonathanRomanucci, ValeriaFarkaš, PavolRomeo, RobertoFarmer, PatrickRomero, Aldo HumbertoFarrell, RobertRomero, AlejandroFarrugia, BrookeRomero-Muñiz, CarlosFasinu, Pius S.Romero-Salguero, Francisco J.Fateixa, Sara Isabel AugustoRondeau-Gagné, SimonFatouro, DimitrisRoot, MartinFaure, SébastienRosa, Marcelo B. DaFaustino, M. Amparo F.Rosato, AntonioFavas, Paulo J.C.Roşca, Sorin-ClaudiuFavero, GaiaRoscini, LucaFavier, IsabelleRose, Peter.Favre-Réguillon, AlainRosebrock, Adam P.Feas, XesusRoselló, SalvadorFedorov, AlexeyRosencrantz, Ruben R.Feiner, Zachary S.Rosicka-Kaczmarek, JustynaFeldblyum, Jeremy I.Rosini, ElenaFelgueiras, HelenaRoss, IanFeliciano, Gustavo TroianoRoss, Ian LFeliu, LidiaRossi, MiriamFenaille, FrancoisRossignol, JulienFeng, Chia-HsienRossiter, SharonFeng, ZhihuiRosso, NataliaFeng, ZhiweiRoszak, SzczepanFenyvesi, FerencRoterman, IrenaFeregrino-Pérez, Ana A.Roth, Stephan V.Ferguson, BradleyRouhiainen, AriFerguson, MatthewRoullier-Gall, ChloéFernadez-Bolanos, José G.Roussel, ChristianFernandes, EduardaRoussis, VassiliosFernandes, P.Routray, WinnyFernández, BelénRoy Choudhury, SwarupFernández, GustavoRoy, DipankarFernandez-Botran, RafaelRoy, RenéFernández-Lafuente, RobertoRoy, SagarFernandez-Muino, MiguelRoychowdhury, SanjoyFernández-Zertuche, MarioRóżalska, BarbaraFerraboschi, PatriziaRozalski, MarcinFerrante, AntonioRozes, NicolasFerrante, ClaudioRozhon, WilfriedFerreira, JorgeRozumová, LuciaFerreira, Leonardo G.Ruan, KeFerreira, MarceloRubin, Seth M.Ferreira, Paula M.Rubino, Federico MariaFerreri, CarlaRubiolo, PatriziaFerretti, ElisabettaRubis, BlazejFerretti, ValeriaRudat, JensFerris, WilliamRudd, Sean G.Ferruz-Capapey, NoeliaRuddraraju, Kasi ViswanatharajuFia, GiovannaRudick, JonathanFigadere, BrunoRudnicka, WiesławaFigueira, Rita BacelarRudrawar, SantoshFigueiredo, Ana Cristina SRudyak, Valery Ya.Figueroa-López, Alejandro MiguelRudzińska, MagdalenaFijan, SabinaRuelland, EricFilannino, PasqualeRuggieri, FabrizioFilarowski, AleksanderRugnini, LorenzaFilek, MariaRui, YueFilho, Edson C. Da SilvaRuiu, LucaFilipova, IneseRuiz Ramos, EncarnaciónFilippov, AndreiRuiz Rubio, LeireFill, Taícia PachecoRuiz, M. PilarFilosto, MassimilianoRuiz, SergioFina, EmanuelaRusinska-Roszak, DanutaFine, James BurkeRusnati, MarcoFinšgar, MatjažRusso, Gian LuigiFiorati, AndreaRusso, MariaFioravanti, StefaniaRusso, MariateresaFiorino, FerdinandoRusso, Mario VincenzoFirlak, MelikeRusso, NinoFisher, Jed F.Russu, WadeFisslthaler, BeateRusu, AuraFleischer, IvanaRuthstein, SharonFletcher, Nicola F.Rutjes, Floris P.J.T.Fletcher, StevenRuzek, DanielFleurat-Lessard, PaulRuzik, LenaFlieger, JolantaRuzza, PaoloFloresta, GiuseppeRyan, AliFodor, CsabaRyan, BarryFodor, MariettaRyan, Edward T.Fogolari, FedericoRyan, Michael P.Fologea, DanielRybarczyk, PiotrFong, Bertram Y.Rybczyńska-Tkaczyk, KamilaFontaine, ThierryRychtera, MojmirFontanesi, ClaudioRyl, JacekFontàs, ClàudiaRyu, Shi YongFontés, MichelRzepecka-Stojko, AnnaForay, GenevieveRzymowska, JolantaForbes, TamaraSá, JacintoFormela, KrzysztofSaad, HosamFormisano, CarmenSabate, RaimonForssell-Aronsson, EvaSabater I Serra, RoserFortes, Ana MargaridaSabatier, Jean-MarcForti, LucaSabella, ErikaFossa, PaolaSabot, CyrilleFoster, TimSacchetti, AlessandroFotopoulos, VasileiosSacks, GavinFoubelo, FranciscoSackus, AlgirdasFouillaud, MireilleSada, KazukiFouillen, LaetitiaSadowska, BeataFourmentin, SophieSaez, CarmenFourmigue, MarcSagui, CelesteFourtounas, CostasSaha, MahaswetaFousteris, ManolisSahi, Vaidurya PratapFox, Jay W.Sahoo, Pabitra KumarFraaije, MarcoSaiano, FilippoFraile, AlbertoSaielli, GiacomoFranca, Eduardo FSainlos, MatthieuFrancesco, NegroSaisho, YoshifumiFrancesco, StellatoSaito, GenkiFrancis, Ashwanth ChristopherSaito, YoshinoriFranco, Albina Cristina RibeiroSaito, YoshiroFranco, Carlos M.Saitoh, KenjiFranco, LourdesSaiz-Fernández, IñigoFranco, RodrigoSakai, AtsushiFrank, Craig L.Sakai, RyuichiFrank, MichaelSakamoto, KensakuFrank, SašaSakilam, SatishFrank, SchweizerSakudo, AkikazuFranko, AndrasSakurai, MinoruFrankowski, MarcinSakurai, ToshihiroFranus, WojciechSala, RobertoFranz, ChlodwigSalachna, PiotrFranzoi, MarcoSalaheen, SerajusFranzyk, HenrikSalahub, DennisFratianni, FlorindaSalas, CarlosFrau, JuanSalazar-Olivo, LAFrazzi, RaffaeleSaldanha, SabitaFrederix, P.W.J.M. (Pim)Salehin, MohammadFreire, JuanSalehizadeh, HosseinFreires, Irlan AlmeidaSalerno, LoredanaFreitas, AnaSales, CarlosFreund, NadjaSalim, VonnyFrezza, ClaudioSalmerón, IvanFriel, ClaireSaluk-Bijak, JoannaFrielinghaus, HenrichSalvador, J.-PabloFrings, PatrickSalvatore, PepeFrkanec, RužaSalvo, AndreaFrochot, CelineSamaja, MicheleFroeyen, MatheusSamanidou, Victoria FFroeyen, MathyŠamec, DunjaFröhlich, EleonoreSamek, OtaFroldi, GuglielminaSamková, EvaFrolov, AndrejSampaio, Natalia G.Frolova, LiliyaSams, ThomasFrongia, AngeloSamuel, TemesgenFrontera, AntonioSan Fabian, EmilioFrosini, MariaSana, Thibault GFu, JunjieSancenón, FélixFu, LiSanchez, Diego H.Fu, PengSánchez, GoarFu, Shih-FengSánchez, María TeresaFu, XueyanSánchez-Fidalgo, SusanaFu, YuSánchez-García, DavidFuglsang, Anja ThoeSánchez-Martínez, ConchaFuhrmann, GregorSanchez-Moreiras, AdelaFujie, TomoyaSancineto, LucaFujihara, TetsuakiSanda, TakaomiFujii, HideakiSandasi, MaxleeneFujii, KazukoSandjo, Louis PergaudFujii, SatoshiSándor, GondaFujimori, KoSandri, Brian J.Fujimoto, KenzoSan-Félix, Ana RosaFujimura, YoshinoriSanganyado, EdmondFujisawa, TomotsumiSangiovanni, EnricoFujita, MasakiSani, GabrieleFujita, MorifumiSanjust, EnricoFujiwara, HidekiSankaran, Ganapathy SubramanianFujiwara, RyoichiSankaranarayanan, Nehru VijiFukuda, MitsuruSanmartín Grijalba, CarmenFukuhara, ToshiyukiSanmartin, ChiaraFukunaga, KenjiSanoh, SeigoFukushi, KeiichiSansone, AnnaFukushima, TomohiroSantacroce, LuigiFuller, AmeliaSantamaria, RitaFumagalli, LauraSantambrogio, PaoloFumanal, MariaSantana, Andrés GonzálezFunabiki, KazumasaSantander-Jiménez, SergioFuoco, TizianaSantangelo, CarmelaFurneri, Pio MariaSanthakumar, AbishekFurness, JamesSantibanez-Lopez, Carlos E.Fürnsinn, ClemensSantilio, AngelaFurtula, BorisSantini, AntonelloFuse, ShinichiroSantolla, Maria FrancescaFuso, FrancescoSantoro, FabrizioFutai, MasamitsuSantos Júnior, Aníbal De FreitasG Gamage, DilaniSantos, ClementinaG. Guirao, Juan LuisSantos, HelderGabdulkhakov, AzatSantos, João M.Gábor, VasasSantos, Marcone MoreiraGaczynska, MariaSantos, Maria M. M.Gafencu, AncaSantos-Gallego, CarlosGaffet, EricSantoso, NettyGagat, MaciejSantulli, GaetanoGăină, Luiza IoanaSanz Garcia, AndresGakiere, BertrandSanz García, JuanGalabov, AngelSaotome, MasaoGalan, CarmenSaoud, KhaledGalan, Javier SanchezSapino, SimonaGalán, Miguel LaderoSarabia, FranciscoGalanis, AlexisSardeli, ChrysanthiGalasiti Kankanamalage, AnushkaSarkar, DhrubaGalat, AndrzejSarkar, SujanGaldeano, CarlesSarma, SauravGaleazzi, RobertaSarti, ElenaGalic, AnteSartori, LucaGálik, BranislavSasai, YasushiGalindo, AntonioSasaki, HideoGalindo-Murillo, RodrigoSasaki, KazuhiroGall Troselj, KoraljkaSasaki, MotokoGallardo, MercedesSasaki, NobuhiroGallemí, MarçalSashiwa, HitoshiGalletti Raspolli, Anna MariaSassi, MohamedGaluska, Sebastian P.Sassone, JennyGalván-D’Alessandro, LeandroSatake, MasayukiGalvez Llompart, MariaSatbhai, Santosh BalasahebGambino, GiorgioSatheesh, SathyanesonGambuti, AngelitaŠatínský, DaliborGamet-Payrastre, LaurenceSato, EmikoGan, RenyouSato, HideyukiGan, Ren-YouSato, HisakoGan, SamuelSato, KazuyukiGan, Yong X.Sato, ShinGandhi, Neha S.Satou, TadaakiGanesan, A.Saturnino, CarmelaGanesan, PalanivelSatyal, PrabodhGangaraju, RajashekharSaucier, CedricGangula, PanduSaurav, KumarGannett, Peter M.Saurina, JavierGantenbein, BenjaminSavage, Paul B.Gao, JianzhaoSavastano, SilviaGao, JieSavi, MoniaGao, PeiyuanSaville, DorothyGao, WenyangSaviola, AnthonyGao, XiaolongSavion, NaphtaliGao, XiaoyanSavoie, Jean-MichelGao, ZheSavy, DavideGarad, SudhakarSawai, HirofumiGaraj, VladimírSawicki, RafalGarami, AndrasSayre, Casey L.Garbowska, MonikaSbardella, GianlucaGarcía De Frutos, PabloScampicchio, MatteoGarcia Estévez, IgnacioScarafoni, AlessioGarcía López, VíctorScarfato, PaolaGarcia Vaquero, MarcoScarselli, MarcoGarcía-Beltrán, OlimpoScarso, AlessandroGarcia-Bosch, IsaacScendo, MieczyslawGarcía-Fresnadillo, DavidSchaefer, JenniferGarcía-Garrido, Sergio E.Schaefer, Jeremy SGarcia-Gimenez, DoloresSchallmey, AnettGarcía-Iriepa, CristinaSchang, LuisGarcia-Jares, CarmenScheicher, Ralph H.García-Moreno, ValmeScheiner, SteveGarcia-Pardo, JavierSchenk, MichaelGarcia-Segura, SergiSchepmann, DirkGarcia-Sosa, Alfonso T.Schiavone, MarcoGarcía-Villalón, Angel LuisSchild-Poulter, CarolineGard, PaulSchillaci, DomenicoGarg, AkhilSchiraldi, David A.Garinis, GeorgeSchirhagl, RomanaGarno, JayneSchirmer, BastianGaro, ElianeSchlueter, Klaus-DieterGarrote, GilSchmeda-Hirschmann, GuillermoGartia, ManasSchmid, JochenGasbarri, CarlaSchmidt, BernhardGase, KlausSchmidtke, MichaelaGašić, Uroš M.Schmitz, John C.Gasparrini, MassimilianoSchnackenberg, LauraGaston, KirkSchneekloth, JohnGatti, AntoniettaSchneider, SabineGauden, Piotr A.Schomburg, LutzGaul, DavidSchrittwieser, StefanGauld, James W.Schroeder, GrzegorzGaumann, AndreasSchuehly, WolfgangGavara, LaurentSchulz, Benjamin L.Gavara, RafaelSchulz, FlorianGawlik-Dziki, UrszulaSchulze, JohannesGawron-Skarbek, AnnaSchulze, MargitGdaniec, ZofiaSchulzke, CarolaGdula-Argasinska, JoannaSchuman, Meredith C.Ge, DongxiaSchurgers, Leon J.Ge, LiyaSchurig, VolkerGe, XiaodongSchwalbe, Carl H.Ge, YufengSchwaminger, SebastianGebicki, JanuszSchwan, MarinaGeissler, DanielSchwan, WilliamGeletii, YuriiSchwartz, Andrew J.Gellerman, GarySchwarze, MichaelGelli, AngieSchweigkofler, WolfgangGelpi, Josep LluisSchweizer, ThomasGemma, SandraSchwiebs, AnjaGemmill, TrentSchwikkard, SianneGeng, AnliŚcianowski, JacekGeng, FengŚcibisz, IwonaGeng, JunlongScicchitano, PietroGenisheva, ZlatinaScognamiglio, MonicaGenova, TullioScorziello, AntonellaGenta-Jouve, GrégoryScott B., HalsteadGentile, MarialuisaScotti, MarcusGentile, PiergiorgioScotti, NunziaGentili, AlessandraScotto D'Abusco, AnnaGeorghiou, ParisSeah, Stephen Y. K.Georgiades, SavvasSeantier, BastienGeorgiev, AntonSebastià, NatividadGeorgiev, GeorgiSebastiana, MónicaGeorgiev, VasilSebestik, JaroslavGeorgieva, RadostinaSeca, AnaGeorgopanos, ProkopiosSeca, Ana Maria Loureiro DaGeraghty, PatrickSechi, GianPietroGerardi, CarmelaSedic, MirelaGérardin-Charbonnier, ChristineSee, Hong HengGerasimova, Yulia V.Segarra-Marti, JavierGerber, Leonard E.Segura, JoanGerdol, MarcoŠegvić Klarić, MajaGereke, ThomasSeidel, Rüdiger W.Gerke, JörgSeidel, ThorstenGerken, MichaelSeiersten, MarionGeronès, Ferran FeixasSeijas Vázquez, JulioGerothanassis, Ioannis P.Seiler, Magdalene J.Gersen, HenkjanSeiner, Brienne N.Gervasi, TeresaSeiquer, IsabelGervasoni, JacopoSękowski, SzymonGessner, GuidoSeley-Radtke, KatherineGhandi, KhashayarSellayah, DyanGhani Zadeh, HosseinSelleri, SilviaGhanotakis, Demetrios FSelvam, ChelliahGhavami, MohammadSelyanchyn, RomanGheni, Ahmed A.Semenyuk, PavelGheorghe, MarianSemeril, DavidGhiasvand, AlirezaSen, SanghamitraGhica, Mihaela-VioletaSenbonmatsu, TakaakiGhislat, GhitaSendker, JandirkGhosh, SantoshSenesi, PamelaGhosh, SubrataSengupta, BidishaGiacomelli, ChiaraSeok, HyunGiacometti, JasminkaSeparovic, FrancesGiacomini, DariaSeptembre-Malaterre, AxelleGiacomino, AgneseSergi, ConsolatoGiacosa, SimoneSerpersu, Engin H.Giampieri, FrancescaSerpico, RosarioGiannakas, ArisSerra, StefanoGiannakoudakis, DimitriosSerradji, NawalGiannakouros, ThomasSerralheiro, Maria LuísaGiannakourou, Maria C.Serrano, MariaGiannenas, IliasSestili, SaraGiarratana, FilippoSethi, GautamGiebułtowicz, JoannaSeto, ShigekiGieling, Roben G.Setzer, William N.Gierlinger, NotburgaSevcovicova, AndreaGierszewska, MagdalenaSeweryn, ArturGiesbertz, Pieter J.Sgherri, CristinaGigoux, VéroniqueSgorbini, BarbaraGikas, EuaggelosSgrignani, JacopoGil, BarbaraShafiei, AliGil, MinchanShah, ManishGilabert-Oriol, RogerShahidi, FereidoonGiles, Wayne R.Shang, ZhuoGilheany, Declan G.Shankar, EswarGill, Harsimran K.Shanker, AnilGilli, FrancescaShao, FangweiGil-Ramirez, GuzmanShao, HuaiyuGilroyed, BrandonShao, YihanGinés Benito, Martínez HernándezSharma, BhavyaGinex, TizianaSharma, BheshGioiello, AntimoSharma, DeepikaGiomi, DonatellaSharma, RitinGiordano, DanielaSharma, RohitGiordano, LuanaSharma, SanjivGiorgi, GiorgioSharman, Edward H.Giosafatto, Concetta Valeria LuciaSharp, JoGiovannucci, David R.Shaughnessy, KevinGirelli, Chiara RobertaShavandi, AminGismondi, AngeloShavrukov, YuriGiuffrè, Angelo MariaShaw, OdetteGiuntini, FrancescaSheardy, RichardGiurg, MirosławShearer, MartinGiuseppina, AndreottiShehu, AmardaGłąbska, DominikaSheldon, JulieGładkowski, WitoldShen, GuofangGladyshev, MichailShen, Szu-ChuanGlaeser, JessieShen, TongyeGlasovac, ZoranShenderovich, Ilya G.Globisch, DanielSheng, RuilongGlover, Stephen A.Sheppard, Tom D.Gmeiner, William H.Sherer, NathanGmurek, MartaSheri, MadhuGnanamani, Muthu KumaranSherwood, JamesGnavi, GiorgioShestopalov, Alexander MGniewosz, MałgorzataShewale, SwapnilGnyba, MarcinShi, Da-YongGobeaux, FrédéricShi, JunfengGobis, KatarzynaShi, SuanGodlewska-Żyłkiewicz, BeataShi, WenboGodlewski, SzymonShi, XiaodongGoel, PeeyushShi, XiaoyangGoettig, PeterShi, XinghuaGoffart, SteffiShi, YiGogulamudi, Venkateswara ReddyShi, ZhenzhenGohda, EiichiShiao, Young-JiGok, OzgulShibata, NorioGolczak, MarcinShiea, JentaieGold, BenShieh, Tzong-MingGolding, JonShigdar, SarahGolkowski, CzeslawShih, Ming C.Goltz, Douglas M.Shiina, TakashiGolubkina, Nadezda AleksandrovnaShikov, Alexander N.Gomes, Maria S.Shil, SuranjanGomes, NelsonShim, Min SukGomes, Nelson G.M.Shimada, MasakoGómez De Cedrón, MartaShimada, YasuhitoGómez García, Carlos J.Shimizu, KuniyoshiGómez, Ana MaríaShimodaira, ShigetakaGómez-Merino, Ana IsabelShimosawa, TatsuoGómez-Pastor, RocíoShin, Beom SooGomez-Plaza, EncarnaShin, DongyunGómez-Ruiz, SantiagoShin, In-SikGomha, SobhiShin, Jae SupGonçalves, Fernando J.Shin, Kwang-HeeGonçalves, Lidia M DShin, Young G.Gonçalves, M. Sameiro T.Shinada, TetsuroGondi, Christopher SShinde, SudhirkumarGonec, TomasShinde, SuhasGoněc, TomášShinichi, SekineGong, XingchuShinomiya, KazufusaGontier, EricShiragami, TsutomuGonzales, Cara B.Shirahata, TatsuyaGonzález Cortés, AraceliShiraishi, TakehikoGonzález Domínguez, RaúlShirakami, YoheiGonzález, Florenci V.Shishido, Tânia K.Gonzalez, Juan MiguelShityakov, SergeyGonzález, María EugeniaShoenfeld, YehudaGonzález-García, JorgeShoji, TsubasaGonzález-Laredo, Rubén FranciscoSholkamy, EmanGonzález-Maeso, JavierShomura, YasuhitoGonzález-Muñiz, RosarioShrimal, ShiteshuGonzález-Sánchez, M. I.Shubina, ElenaGonzález-Stuart, ArmandoShuhei, NasudaGonzález-Vallinas, MargaritaShukla, HemGonzález-Vergara, EnriqueShul'pin, Georgiy B.Gopalan, Sai AnandShults, Elvira E.Gordillo Arrobas, BelénShumskaya, MariaGordon, Michael H.Siano, FrancescoGórecki, MarcinSiatka, TomášGoreham, ReneeSiczek, MiłoszGorgoulis, VassilisSidorenko, Viktoriya S.Gorham, EdwardSiebenhofer, MatthäusGori, AntonellaSieber, FritzGorman, GregSiemer, Ansgar B.Goroncy, AlexanderSienkiewicz, MonikaGorrell, MarkSier, Cornelis F.M.Górska, SabinaSiewert, BiankaGorska-Ponikowska, MagdalenaSigusch, Bernd W.Gorte, Raymond J.Sikazwe, DonaldGościańska, JoannaSikirzhytski, VitaliGosetti, FabioSilaghi-Dumitrescu, RaduGossage, R. A.Silchenko, Alexandra S.Goswami, SubhadipŠiler, BranislavGoszczyński, TomaszSilina, YuliyaGoto, MasuoSilva, Amelia MGotor Fernandez, VicenteSilva, AndreGouany, FradySilva, Artur M. S.Goudarzi, MaryamSilva, Carlos AlbertoGoulas, VlasiosSilva, Dina M.Goupil, Brad ASilva, JoséGoze, ChristineSilva, LuísGozzelino, RaffaellaSilva, Luís R.Gozzo, FrancoSilva, Márcio S.Grabacka, MajaSilva, Margarida F. B.Grabarczyk, MałgorzataSilva, Maria FátimaGrabulosa, ArnaldSilva, Pedro JorgeGradinaru, Andrei CristianSilva, Robson R.Graiff, ClaudiaSilva, Susana N.Grajek, HenrykSilva, Vera L. M.Granados-Principal, SergioSilvagno, FrancescaGranchi, CarlottaSilvestre, SamuelGrande, FedoraSilvestri, RomanoGrande, RossellaSim, ValerieGrandjean, CyrilleSimal-Gandara, JesusGranica, SebastianSimão, DanielGrant, GeorgeSimari, CataldoGrant, WilliamSimeonov, IvanGranzhan, AntonSimeonov, Vasil D.Grasselli, ElenaSimha Martynkova, GrazynaGrassmann, AndréSimirgiotis, MarioGraton, JérômeSimitzis, PanayiotisGray, Christopher A.Simka, WojciechGrazioso, GiovanniŠimko, FedorGreen, CharlotteSimmons, KatieGreen, Ivan RobertSimocko, ChesterGreenberg, ArthurSimoes, MarioGreenhalgh, DavidSimões, Marta FilipaGregáň, JurajSimon, JulianGregory, StephenSimón, LuisGreim, HelmutSimone, Angela DeGrellier, PhilippeSimone, ElenaGrembecka, MałgorzataSimonneaux, GerardGrider, ArthurSimpson, T. J.Griesbeck, AxelSims, Ian M.Griesser, MichaelaSingh, AlokGriffiths, PeterSingh, ArunimaGriga, MiroslavSingh, ChanpreetGrijalvo, SantiagoSingh, Jitendra KumarGrisanti, LucaSingh, JugpreetGrisoni, FrancescaSingh, KamalendraGrivel, Jean-ClaudeSingh, KhushwantGrkovic, TanjaSingh, SarbjitGross, AaronSingh, SashaGrossi, GiancarloSingh, SomnathGrossi, MarcoSingh, Surya P.Grossini, ElenaSingha, SubhankarGrossman, Jerrold W.Singhrao, Sim K.Grosso, GiuseppeSinha, AbhijeetGruber, ChristianSinha, Birandra K.Gruhlke, MartinSinha, JasmineGrunig, GabrieleSinha, KalyanGrützmacher, HansjörgSinha, SudarsonGrynkiewicz, GrzegorzSintim, HermanGrzegorczyk-Karolak, IzabelaSinyukov, AlexanderGrzybowska, EwaSipos, LászlóGu, MengmengSippl, WolfgangGu, ZhenSipponen, Mika HenrikkiGuadagno, CarmelaSips, PatrickGuamis, BuenaventuraSiracusa, LauraGuarcello, RosaSirimulla, SumanGuarino, CarmineSita, GiuliaGubin, DenisSitarek, PrzemysławGudbrandsen, Oddrun A.Sivaev, Igor B.Guerrini, AlessandraSivakumar, SushamaGuha, Prajna P.Sivasubramanian, AravindGuibal, EricSivieri, KatiaGuido, Luis F.Siwek, AgataGuillon, ChristopheSizochenko, NataliaGuiot, CaterinaSkalicka-Woźniak, KrystynaGullón, BeatrizSkalický, MilanGumieniczek, AnnaSkaltsa, Helen D.Gumienna, MałgorzataSkandalis, NikolaosGündisch, DanielaSkellam, ElizabethGündoğ, YücesanSkenderi, Katerina PGunia-Krzyżak, AgnieszkaSkiba, MohamedGuo, FenghaiSkorik, Yury A.Guo, PuSkretas, GeorgiosGuo, ShaoyunSkroza, DanijelaGuo, XiaofengSkwarło-Sónta, KrystynaGupta, KajalSlavik, RogerGupta, SanjaySlipchenko, LyudmilaGupta, Sheeba VargheseŚlipiko, MonikaGupta, VivekSliwiak, JoannaGupta-Rossi, NeetuŚliwińska-Wilczewska, SylwiaGurgul, ArturŚliwiński, TomaszGurikov, PavelŚliwka-Kaszyńska, MagdalenaGursoy, GamzeSłoczyńska, KarolinaGusev, AlexanderSlominski, AndrzejGushina, IrinaSlosarczyk, AgnieszkaGutiérrez-Gallego, RicardoSluchanko, NikolaiGutiérrez-Uribe, JanetSlusarenko, AlanGütschow, MichaelSmani, YounesGuvench, OlgunSmaoui, SlimGuzik, UrszulaŠmejkal, KarelGuzman-Puyol, SusanaSmeriglio, AntonellaGuzow-Krzemińska, BeataSmetana, KarelH. Moreno, Paulo RobertoSmetana, MichaelHabash, MarcSmid, Scott D.Habata, YoichiSmiesko, MartinHabtemariam, SolomonŚmiga-Matuszowicz, MonikaHachemi, ImaneŚmigielski, Krzysztof B.Hackauf, BerndSmith, MarkHackett, MarkSmith, MatthewHackmann, TimothySmith, Micholas DeanHadaruga, DanielSmith, Robert A.Hadjikakou, SotirisSmith, TerryHadjipavlou-Litina, DimitraSmout, MichaelHaenen, GuidoSmrke, SamoHafuka, AkiraSmułek, WojciechHager, Martin D.Smyrniotopoulos, VangelisHaidar, SamerSnegur, LubovHaider, NorbertSnežana, PapovićHaider, ShozebSnowden, TimothyHains, DavidSobeh, MansourHalder, AvikSobiech, MonikaHam, Kyung-SikSobisch, TitusHamaguchi, MasahideSobkowski, MichalHamman, JoiasSobotta, ŁukaszHammann, SimonSobral, Abilio J.F.N.Hammer, KatherineSocaci, SoniaHammerl, Jens AndreSoejima, TetsuroHammerschmidt, RaySoengas, RaquelHan, Byung WooSoengas, Raquel G.Han, JaehongSoica, CodrutaHan, JianlinSokół-Łętowska, AnnaHan, Jong WonSokolov, MaximHan, KewenŠolaja, Bogdan A.Han, MingyuanSolano, FranciscoHan, QifengSolcan, CarmenHan, Quan-BinSolel, EphrathHan, Sung NimSoleymani Abyaneh, HodaHan, WeiweiSoliman, Mahmoud EHan, Won-SikSoliven, ArianneHan, YifanSollogoub, MatthieuHandzlik, JadwigaSolomon, MelaniHaneklaus, MoritzSolomonov, BorisHanganu, DanielaSoloshonok, Vadim A.Hanhineva, KatiSolovyev, AndreyHaniu, HisaoSomani, SukrutHannafon, BethanySommer, StephanHano, ChristopheSon, JaekyoungHanschen, FranziskaSong, ChunhuaHansen, EspenSong, GuoqingHansen, NielsSong, ImsookHanus-Fajerska, EwaSong, JinhuaHao, JijunSong, Kwang HoHappel, AustinSong, Kyung BinHarada, MamoruSong, MeiHarashima, NanaeSong, Sang HoonHarasym, JoannaSong, TaoHarding, IanSong, XuHardingham, Jennifer ESong, Yang-heonHargreaves, Justin Stephen JamesSopade, Peter A.Harijan, Rajesh KŠoral, MichalHariri-Ardebili, Mohammad AminSorg, OlivierHarmanci, Arif OzgunSortais, Jean-BaptisteHaro, DiegoSosna, JustynaHarp, Joel M.Soto-Hernández, MarcosHarper, JamesSoucy, ShannonHarrison, Jonathan S.Soulère, LaurentHarrison, Paul H. M.Soural, MiroslavHarrison, RobertSousa, AnaHarvey-Samuel, TimSouza-Smith, FlaviaHasanuzzaman, MirzaSovova, HelenaHasegawa, MorifumiSowa, IreneuszHashidzume, AkihitoSpanakis, MariosHashimoto, MakotoSpano, GiuseppeHashimoto, MasaruSpanopoulos, IoannisHassan, MohamedSparacino-Watkins, CourtneyHassan, Sherif T. S.Speciale, AntonioHatae, NoriyukiSpee, BartHatami, AsaSpégel, PeterHatano, TsutomuSpinella, AldoHatfield, Ronald D.Spinelli, LauraHatterman-Valenti, HarleneSpingler, BernhardHatzidimitriou, EfimiaSpivak, DavidHaudecoeur, RomainSpohr, EckhardHaugen, Havard JosteinSpradley, Frank T.Hausmann, MichaelSpringer, LindsayHausner, GeorgSprio, Andrea ElioHawrył, Anna M.Spur, Bernd W.Hay De Bettignies, Anne-EmmanuelleSpyros, GkelisHay, MichaelSreedasyam, AvinashHayakawa, TakehitoSreerama, SubramanyaHayashi, FumitakaSridhar, JayalakshmiHayashi, ShotaroSrihari, SriganeshHayashi, YoshihitoSrinivas, KeerthiHayes, K C.Sriram, GopuHayn, RolandSrivastava, AtulHazai, LászlóSrivedavyasasri, RadhakrishnanHazak, OraSrivenugopal, KalkunteHe, HailunSroka, ZbigniewHe, HongliangStaal, JensHe, JieStacchiotti, AlessandraHeaney, LiamStadler, MarcHearn, Michael J.Stadnik, JoannaHeath, TimothyStagos, DimitriosHeber, DieterStaniszewska, MonikaHegedűsová, AlžbetaStankevic, MarekHeider, DominikStanovnik, BrankoHeinbockel, ThomasStarostik, PetrHeiner, DetertStarowicz, MałgorzataHeipieper, HermannStarzak, KarolinaHeise, H.M.Stasiuk, GraemeHeiskanen, JuhaStassen, IvoHelaly, SoleimanStaszak, KatarzynaHeld, ChristophStavrianidi, AndreyHeleno, SandrinaStecca, BarbaraHellwig, MichaelSteck, Todd R.Helm, RichardSteed, JonathanHeltzel, JacobSteele, Terry W.J.Hemraz, Usha D.Stefane, BogdanHenderson, WilliamStefanowicz-Hajduk, JustynaHendriks, WiljanStefanuto, Pierre-HuguesHenen, Morkos A.Stefek, MilanHenkel, JaninSteibliene, VestaHenrique, RuiStein, MatthiasHenry, Ryan A.Steinfeldt, NorbertHensel, AndreasSteinmann, CasperHepnarova, VendulaStenstrøm, YngveHer, SongStepanovic, StepanHerbert, JohnStephan, HolgerHerczegh, PálStephen, Michael RajeshHeredia-Guerrero, José AlejandroStephens, Jacqueline M.Herker, EvaStępień, ŁukaszHerman, Andrzej PrzemysławSteup, MartinHernández, FranciscaStevens, ColeHernández, IgnacioStevens, ErlandHernández-Córdoba, ManuelStevenson, BradleyHernández-Hernández, ÁngelSteverding, DietmarHernández-Mesa, MaykelStieger, BrunoHernando, JordiStilli, DonatellaHerndon, James W.Stipanovic, ArthurHerold, NikolasStiriba, Salah-EddineHerráez-Hernández, RosaStjepanović, MarijaHerrera, Raquel P.Stobiecka, MagdalenaHerrera-May, AgustínStobiecki, MaciejHerrero, BaudilioStöckelhuber, Klaus WernerHerres-Pawlis, SonjaStockmann, ChristianHerrmann, FabianStoikov, Ivan IvanovichHerrmann, MathiasStojakowska, AnnaHerwadkar, AnushreeStojan, JureHester, JoannaStojko, JerzyHideg, ÉvaStoleru, ElenaHiejima, YusukeStone, KariHikawa, HidemasaStonik, Valentin A.Hildén, KristiinaStornaiuolo, MarianoHildenbrand, GeorgStortini, Angela MariaHildreth, BlakeStratford, Robert E.Hildreth, SherryStrati, Irini F.Hill, DanielStratmann, BerndHill, J. GrantStraus, Suzana K.Hillwig, MatthewStrauss, PhyllisHiltunen, MinnaStrehmel, VeronikaHinrichs, Wouter L.J.Strekowski, LucjanHirakawa, HidehikoStreubel, RobertHirasaka, KatsuyaStroffekova, KatarinaHirasawa, NoriyasuStrube, JochenHirayama, FumitoshiStruga, MartaHiroaki, HidekazuStrzelecki, MichalHirota, KiichiStrzemiecka, BeataHirota, YuichiroStuart, DavidHixson, JoshStudzińska-Sroka, ElżbietaHo, Eric S.Stuper-Szablewska, KingaHo, JunmingSturm, SonjaHo, Mei-LinStürup, StefanHo, MengfeiSu, ChunmingHo, Shin-LonSu, Jui-HsinHo, Su-ChenSu, MengHo, Wing ShingSu, PeifengHo, Yi-ChengSubramaniam, PrasadHoang, Nam VSucheck, Steven J.Hocek, MichalSuda, YoshihitoHockey, MeghanSugahara, TakuyaHodge, AllisonSugai, TakeshiHoffman, AngelaSugawara, AkihiroHoffmann, KlausSugimoto, YukioHofmann, JohannSugimura, HaruhikoHofmann, UteSugiura, YoshimasaHoggard, Patrick E.Suh, Dae-yeonHohensinner, PhilippSuhail, YasirHohmann, JuditSukhikh, TaisiyaHolalu, SrinidhiSukumaran, AnilHolder, Alvin A.Sukumaran, SunithaHollenhorst, Peter CSuleria, HafizHolmes, Dawn E.Suliburska, JoannaHolst, OttoSulman, EstherHolynska, MalgorzataSulová, ZdenkaHolzmann, KlausSun, ChenHolzmann, NicoleSun, DianqingHomaeigohar, ShahinSun, FengjieHomma, Miwako KatoSun, GuohuiHonda, MasakiSun, HaoHong, Jin-LongSun, JingjingHong, Mee YoungSun, LinaHong, YanjunSun, LitaoHong, YonggeunSun, MengHong, YuningSun, ShiHonzíček, JanSun, Shih-JyeHöög, JohannaSun, WeiHoogeveen, Ellen K.Sun, WujinHook, IngridSun, XinghuiHooper, CorneliaSun, YingjieHorax, RonnySunami, YoshiakiHorino, YoshikazuSunatsuki, YukinariHoriuchi, YasueSundheim, LeifHorowitz, Rami A.Sung, Ping-JyunHorvai, GeorgeSung, Wang ChouHorvath, DragosSung, Wen-ChiehHoshino, MasaruSuravajhala, PrashanthHosomi, RyotaSuresh, KuthuruHosoya, TakamitsuSurguchov, AndreiHosoya, TakashiSurma, MagdalenaHossan, Mohammad RobiulSurmenev, Roman A.Hosseini, AbolfazlSurmick, DavidHošťálková, AnnaSurup, FrankHostaš, JiříSuryawanshi, VasantikaHotta, Jun-ichiSusic, MichaelHou, Duen-RenSutherland, John B.Hou, HarveySutton, Peter W.Hou, JingweiSuzuka, ToshimasaHougland, JamesSuzuki, KatsuhikoHoung, Jer-YiingSuzuki, KazumasaHour, Mann-JenSuzuki, ShigekatsuHouzé, PascalSuzuki, ToshisadaHowarth, AshleeSuzurikawa, JunHoyos, PilarSvärd, MichaelHrabal, RichardŠvecová, BlankaHrnčič, Maša KnezSveda, MariaHroboňová, KatarínaSvetashev, VasilyHsia, Shih-MinSvete, JurijHsiao, GeorgeSvingen, TerjeHsieh, ChangweiSvob Strac, DubravkaHsieh, Feng-ChiaSvoronos, Paris D.N.Hsieh, Hsi-LungSwarnkar, GauravHsieh, Pei-WenSwevers, LucHsin, Kun-YiŚwiątek, ŁukaszHsu, Fu-YinŚwiątek, PiotrHsu, I-JuiŚwiderska-Dąbrowska, RenataHsu, Kuo-ShengSwierczynski, JulianHsu, Shu-haoSwiezewska, EwaHsu, Sung-PoSydnes, Magne O.Hsueh, Yi-HuangSyed, ViqarHu, BoSylwester, SlusarczykHu, GuangSynytsya, AndriyHu, PingSyrpas, MichailHu, QiwenSzaciłowski, KonradHu, Shang-HsiuSzacon, ElzbietaHu, Wei-PingSzafraniec, JoannaHu, XiaoSzafrańska, KatarzynaHu, YongSzafranski, KrzysztofHu, ZhenbinSzakonyi, ZsoltHuang, Chi-ChangSzaleniec, MaciejHuang, Chih-ChingSzczolko, WojciechHuang, Chih-FengSzekalska, MartaHuang, Chih-LingSzekely, GyorgyHuang, Chiung-ChengSzeleszczuk, ŁukaszHuang, Genin GarySzewczyk, KatarzynaHuang, Guan-JhongSzewczyk, RomanHuang, HaoSzikra, DezsőHuang, Hsu-ShanSzilágyi, AndrásHuang, Hui-ChiSzilvási, TiborHuang, Hung-ChungSzklarczyk, DamianHuang, Jason C.Szkudelska, TomaszHuang, Jiann-JyhSzőri, MilánHuang, JingfengSzostak, MichalHuang, Jui-HsienSztanke, MałgorzataHuang, LeiSzumny, AntoniHuang, LingSzumny, Antoni JacekHuang, Shu-ChiSzweda, PiotrHuang, Wei-JanSzymanowska, UrszulaHuang, Wen-ChungSzymańska, EmiliaHuang, Wen-LiiSzymański, PawelHuang, WenlinTa, HangHuang, XiaoTabarrini, OrianaHuang, XueshiTabarsa, MehdiHuang, YuchengTabata, MakotoHuang, YufengTacconelli, StefaniaHubková, BeátaTadanaga, KiyoharuHübner, FlorianTadashi, WatabeHuck, ChristianTaddei, MaurizioHuczyński, AdamTadepalli, SirimuvvaHudson, RobertTadic, VanjaHuefner, AntjeTagliatesta, PietroHueso, Jose L.Taguchi, Y-h.Hughes, Richard K.Tahir, Muhammad NHuh, Do SungTai, Dar-FuHuh, Jae-WonTaïeb, DavidHuh, Yun SukTaira, JunseiHumpl, TilmanTajber, LidiaHung, Che-LunTakabe, WakakoHung, Kuo-HsiangTakada, TomoyaHungerford, JamesTakagi, YasumasaHuot, PhilippeTakahama, UmeoHuppi, KonradTakahashi, DaisukeHur, Sun-JinTakahashi, FumikiHurley, MargaretTakahashi, KaitoHussein, WaleedTakahashi, KosakuHusson, JérômeTakahashi, OhgiHuszcza, EwaTakahashi, OsamuHutter, MichaelTakahashi, ShoujiHwang, Bang YeonTakahashi, TakuHwang, Long-ChihTakahashi, ToshioHwu, Jih-RuTakanami, ToshikatsuHye, Jin JungTakano, KatsuraIacopetta, DomenicoTakayama, YoshiharuIacovino, RosaTakayanagi, ToshiyukiIadarola, PaoloTakayuki, BanIannazzo, DanielaTakebayashi, Shin-ichiroIanora, AdriannaTakeda, YouheiIatsunskyi, IgorTakemori, HiroshiIbeas, JoséTakemoto, HiroyasuIbrahim, Salam ATakemoto, MasumiIchimura, KazuyaTakemura, MihoIentile, RiccardoTakenaka, ShinjiIglesias, Luis E.Takeshita, HaruoIguchi, TaisenTakeuchi, DaisukeIhle, AndreasTakizawa, ShinobuIijima, NoriakiTakó, MiklósIjima, HiroyukiTakulder, PoulamiIkegami, TakahisaTalele, Tanaji TIkehara, KenjiTalhi, OualidIlagan, Ma. Xenia G.Talora, ClaudioIlarraza, RamsesTaly, AntoineIleana Ionescu, MihaelaTam, Eric Kwok WaiIlia, GheorgheTamai, IkumiIlie, Ioana M.Tamama, KenichiIliev, BoyanTamm, ToomasIlisz, IstvánTamminen, TarjaIlla, OnaTampucci, SilviaImaz, InharTamura, OsamuImhof, PetraTan, Howe-SiangImig, JohnTan, Ming-QianImm, Jee-YoungTan, ZhongpingImokawa, GenjiTanabe, KatsuyukiImperatore, ConcettaTanaka, HiroyukiImperlini, EstherTanaka, KazuoInaba, HiroshiTanaka, NaonobuInagaki, HidetoshiTanaka, NobuyukiInagaki, NaokiTanaka, ShigenoriInagaki, NoritoshiTanaka, ShinjiInaguma, ShingoTanaka, TakayukiInamura, KentaroTanaka, TomonariIndiveri, CesareTanase, CorneliuInfante, PaolaTanemura, KiyoshiInfantes Molina, AntoniaTang, HouliangIngram, ConradTang, LongtengIngrosso, FrancescaTani, FumitoInguimbert, NicolasTani, HiroumiInngjerdingen, MaritTaniguchi, NobukazuInokuchi, TsutomuTaniguchi, TohruInoue, AtsukoTaniguchi, YasushiInsenser, MaríaTanimoto, HirokiInuzuka, ToshiyasuTankiewicz, MaciejIoelovich, Michael YaTanner, EdenIonita, PetreTanner, Julian A.Ionut, Ioana A.Tantillo, DeanIorizzi, MariaTao, LinIossifidou, EleniTao, PengIqbal, Hafiz M.N.Tapia, Alejandro A.Irakli, MariaTarantino, GiovanniIram, SurtajTarman, KustiariyahIriepa Canalda, IsabelTarozzi, AndreaIsaza Martínez, José HipólitoTashima, KimihitoIsemura, MamoruTashima, ToshihikoIshigaki, MikaTashiro, EtsuIshii, TakahiroTashiro, HirotakaIskierko, Zofia EwaTasic, LjubicaIslam, M. NurulTasmin, SairaIslam, Md SorifulTate, Rothwelle J.Ismael, M. IsabelTatikolov, Aleksander S.Ismaili, LhassaneTatsumi, YukiIsman, Murray B.Taube, JoeIsovitsch, RalphTaura, FutoshiIta, KevinTava, AldoIto, HideyukiTavares, AnthonyIto, Jun-ichiTavares-da-Silva, Elisiario JoséIto, ShingoTayebati, Seyed KhosrowIto, TakeruTaylor, CarolineIto, TakuyaTaylor, MartinIto, YoichiroTaylor, MatthewItoh, TakafumiTaylor, Maureen E.Itoh, ToshiyukiTcherdyntsev, Victor V.Itou, JunjiTecce, Mario FeliceItsuno, ShinichiTedesco, IdoloIturriaga-Vasquez, PatricioTeeri, TeemuIvancevic, AtmaTeixeira Tomaz, CândidaIvanov, Alexander VTeixeira, RóbsonIvanov, AndreyTelegdi, JuditIvanov, Sergey M.Tellez, Trinidad RuízIvanova, BojidarkaTelukutla, Srinivasa ReddyIvanova, YordankaTelysheva, GalinaIwai, AtsushiTembrock, Luke R.Iwamoto, SatoshiTempone, AndréIwamoto, ShotaroTeng, BoIwanicki, AdamTeng, YongIwasa, TakeshiTengfei, JiIwasaki, TakashiTeodorescu, FlorinaIwatsuki, MasatoTeodoro, JoãoIzadyar, AnahitaTepe, Jetze J.Izgu, Enver CagriTerčelj, MarjetaIzquierdo-Rico, Maria JoséTerent'ev, Alexander O.Izsvák, ZsuzsannaTerenzi, AlessioIzumi, YudaiTerpou, AntoniaJabłońska-Trypuć, AgataTerry, Randall G.Jablonski, MiroslawTerzaghi, WilliamJackson, Michael A.Terzaghi, William BryanJackson, William F.Teschke, RolfJacob, S. JohnTetlow, Ian J.Jacobsen, Elisabeth EgholmTeusch, NicoleJacquemin, DenisTeymourian, HazhirJadeja, Ravirajsinh NavalsinhThakkar, BalmukundJadhav, Gopal P.Thakur, Vijay KumarJaenicke, StephanThamattoor, Dasan M.Jaffrezic-Renault, NicoleThapar, RoopaJafra, SylwiaThiéry, ValérieJagerovic, NadineThilmony, RogerJagiello, KarolinaThirunavukarasu, DeepakJahandideh, SamadThiruvengadam, MuthuJahidul, IslamThissen, PeterJain, AkshayThomas, Eric J.Jaiswal, Amit K.Thomas, Eric JimJaiswal, JagdishThomas, JoiceJaiswal, RakeshThomas, Paul M.Jäkle, FriederThomas, SamuelJakóbik-Kolon, AgataThomas, T.J.Jakopic, JernejaThomas, Y. K. ChanJakubczyk, AnnaThomson, Neil H.Jakubikova, JanaThreadgill, MichaelJalkanen, Aaro J.Thyagarajan, BaskaranJames, PaulTiera, MarcioJamieson, AndrewTietjen, IanJamieson, Stephen M. F.Tietze, AlesiaJampaiah, DeshettiTiffen, Jessamy C.Jamroz, MichalTilley, MichaelJana, NavenduTimón, VicenteJanakiraman, HarinarayananTimoshenko, Alexander V.Janczarek, MonicaTimperio, Anna MariaJandrić, ZoraTing, Richard C. H.Jang, Byoung KukTiruveedhula, V. V. N. Phani BabuJang, Dae SikTischler, DirkJanissen, RichardTithi, Jesmin JahanJankauskaite, VirginijaTiu, Brylee DavidJanovák, LászlóTiwari, AmitJanowski, MiroslawTiwari, Rakesh K.Jansen, RolfTiwari, VaibhavJantas, DanutaTo, Kin-YingJaradat, NidalTobrman, TomášJarmoluk, AndrzejTocmo, RestitutoJarosz-Wilkolazka, AnnaToczylowska-Maminska, RenataJarret, Robert L.Todde, SergioJarrous, NayefTodea, AnamariaJasicka-Misiak, IzabelaToiu, AncaJasik, PatrykTojo, ConchaJasinski, MarcinTolosa, EzequielJasiński, RadomirToma, LucioJaumot, JoaquimTomalia, DonaldJavier, Sánchez-NievesTomalia, Donald A.Jayakumar, RangasamyTomar, DhanendraJayakumar, ThanasekaranTomašič, TihomirJayamohan, HarikrishnanTomaszewska, EwaJayant, Rahul DevTomczyk, MichałJe, Jae-YoungTomczykowa, MonikaJean, LudovicTomi, FélixJeandet, PhilippeTomoda, HiroshiJeandroz, SylvainTomuta, IoanJedynak, KatarzynaTondi, GianlucaJee, JunGooTonelli, MicheleJelen, HenrykTonolo, GiancarloJelliss, PaulTopic, FilipJena, Prasant KumarTori, MotooJenett-Siems, KristinaToribio, LauraJenkins, IanTorigoe, KanjiroJenkins, Samir V.Torikai, KoheiJennings, Jessica AmberTormo, José RubénJensen, Lars HenrikTorre, Maria LuisaJenssen, HåvardTorres Lagares, DanielJeon, Byeong HwaTorres Perales, CarolinaJeon, HyeonyeolTorres Vaamonde, Jose EnriqueJeon, Noo LiTorres, JaumeJeon, Sung HoTorres, Sara SopeñaJeong, DaewonTorres-Chávez, Patricia I.Jeong, Heon-HoTorroba, TomásJeong, Ki JunTortorella, DomenicoJeong, Lak ShinTósaki, ÁrpádJeong, Seon-YongToscani, AnitaJeong, Soo HeeTosheva, LubomiraJeremy, Teoh Yuen ChunToshio, KoizumiJerković, IgorTosi, SimoneJerome, De RuyckTosoni, SergioJeschke, GunnarTóth, BlankaJetten, AntonTóth, GergőJha, MithileshTotsingan, FilbertJi, TianjiaoTotta, PierangelaJiang, DianluTotti, FedericoJiang, JiangTouil-Boukoffa, ChafiaJiang, JunkeToy, RandallJiang, XuntianTrabace, LuigiaJiang, Yuan-YeTrabocchi, AndreaJiang, ZaixingTrainor, PatrickJiao, YinpingTrammell, Scott A.Jimbo, MitsuruTramontano, EnzoJimenez, M. AngelesTran, FabienJimenez, RandiTran, PhuongJiménez-Alemán, GuillermoTrandafirescu, CristinaJiménez-Araujo, AnaTranfo, GiovannaJiménez-Aspee, FelipeTrant, JohnJimenez-Barbero, JesusTravers, Jeffrey BryantJimenez-lopez, Jose C.Traweger, AndreasJin, KailongTrentini, AlessandroJin, ZhaoTrépout, SylvainJindrich, JindrichTrevisan, AndreaJirovetz, LeopoldTribe, LorenaJo, Young SukTriebl, AlexanderJoão Ramalhosa, MariaTrif, MonicaJoão, BenevidesTrinchera, MarcoJohn, ŁukaszTrincone, AntonioJohnson, DavidTrindle, CarlJokić, StelaTripathi, AshootoshJolly, PawanTripathi, Lokesh P.Jones, ChrisTripathi, ManishJones, DavidTripodi, GianlucaJones, Kathryn MarieTripodo, GiuseppeJones, MarjorieTripp, Carl P.Jones, MerielTríska, JanJonsson, SofiTrivedi, Evan R.Joo, Hong-GuTroise, Antonio DarioJorda, RadekTrojanowicz, BoguszJordão, António ManuelTrombetta, DomenicoJordao, LuisaTrotsko, NazarJørgensen, KåreTrougakos, IoannisJose, JineyTrovato, FabioJoshi, HirenTruhlar, Donald G.Joshi, Pushpa RajTrujillo, Antonio-JoséJoshi, SanketTrujillo, CristinaJoubert, FannyTrzeciak, Anna M.Joule, John A.Tsafantakis, NikolaosJovanović, DraganaTsai, Ang-ChenJovanovic-Santa, SuzanaTsai, Teh-huaJoven, JorgeTsai, YenlingJówko, EwaTsaltas, DimitriosJu, Lu-KwangTsapogas, PanagiotisJuang, Shin-HunTsay, Chien-YieJudith, María JudithTse, William K.F.Judson, Richard STseng, Chih-HuaJugessur, AjuTseng, Wei-LungJuhasz, BelaTsirigos, KonstantinosJulianna, OláhTsitsilonis, Ourania E.Juliano, ClaudiaTsoi, JamesJullian, NathalieTsopmo, ApollinaireJullien, FrédéricTsotinis, AndrewJung, Chol-HeeTsubaki, KazunoriJung, JaehanTsuchida, SachioJung, SeunhoTsuchimoto, TeruhisaJunior, Ary FernandesTsuchiya, KousukeJuodkazis, SauliusTsuji, Atsushi B.Jurado, Amalia S.Tsuji, HiroakiJurado-Sánchez, BeatrizTsujibo, HiroshiJurić, MarijanaTsukamoto, MasakiJurikova, TundeTsunoda, MakotoJurman, GiuseppeTsuruoka, TakaakiJuskowiak, BernardTsutsui, ShigeyukiJusticia, JoséTsutsumi, OsamuJuszczak, LesławTsyganenko, Alexey A.Juvik, JohnTsyshevskiy, RomanK A Foyez, MahmudTu, MinK. Hashmi, StephenTuccinardi, TizianoKabała-Dzik, AgataTucker, StevenKabelác, MartinTudela, JoséKabil, OmerTuenter, EmmyKaczor, Agnieszka A.Tueros, ItziarKaczorek, EwaTulkens, PaulKadela-Tomanek, MonikaTullai, JohnKadokawa, Jun-ichiTullio, VivianKadota, IsaoTullius Scotti, MarcusKafarski, PawelTunaru, SorinKafle, LekhnathTunesi, MartaKahlert, HeikeTuominen, AnuKahr, BartTupally, Karnaker R.Kaiser, LeonardTurano, PaolaKaiser, MarcelTurchin, IlyaKaja, SimonTurčić, PetraKajimoto, ShinjiTurco, RosaKajiya, HiroshiTurel, IztokKakkar, AshokTurel, MatejkaKalai, TamasTurk, TomKalani, KomalTurkman, NashaatKalayda, GannaTurkowski, VolodymyrKalemba, DanutaTurrini, EleonoraKalhapure, Rahul S.Tuti, SimonettaKali, GergelyTuttolomondo, AntoninoKaliniewicz, ZdzisławTuvdendorj, DemidmaaKalinin, VladimirTuzimski, TomaszKalinowska Lis, UrszulaTvedt, Tor Henrik AndersonKalinowska, MonikaTvrdeic, AnteKallifidas, DimitrisTvrdik, PetrKalogiannis, StavrosTyagi, RahulKalogirou, Andreas S.Tzakos, Andreas G.Kalsi, MeghaTzen, Jason T.Kaltner, HerbertTzounis, LazarosKamagata, KiyotoTzschucke, ChristophKambhampati, Siva PramodhUccelli, LiciaKamdem, Donatien PascalUccello-Barretta, GloriaKamdem, Jean PaulUchio, YujiKamel, Karolüchncü, MuhammedKameo, HajimeUddin, ShaikhKameyama, AtsushiUdumula, Venkata ReddyKamiloglu, SenemUeda, HiroshiKamitori, ShigehiroUeda, MitsuhiroKamzolova, SvetlanaUehara, TomoyaKan, Chi-waiUekusa, HidehiroKanamori, TakaoUeng, Yune-FangKananovich, Dzmitry G.Ueno, TakafumiKanaujia, ParijatUeta, IkuoKanazawa, ArihiroUfnal, MarcinKanbara, TakakiUhde, ErikKanekiyo, TakahisaUhde-Stone, ClaudiaKanetis, LoukasUitdehaag, JoostKang, Chang HoUivarosi, ValentinaKang, CongBaoUjor, Victor C.Kang, Hee Y.Ulanski, PiotrKang, Hui-SeungUlasov, IlyaKang, Hyun WookUludag, HasanKang, Jae SeungUmemori, JuzohKang, Jeon WoongUmerska, AnitaKang, JonghoonUmeyama, AkemiKang, Jun YongUmezawa, TaikiKang, KyungsuUmoren, SaviourKang, SinyoungUneyama, TakashiKangas, MichaelUnno, KeikoKano, NaokiUno, HidemitsuKantminienė, KristinaUno, TomohideKanzaki, HiroyukiUnsworth, WillKao, Chai-LinUnterlass, Miriam M.Kao, Erl-ShyhUnterreiner, Andreas-NeilKaparaju, PrasadUnudurthi, Sathya DevKapusta-Duch, JoannaUpadhyay, Rakesh K.Kar, AdwitiyaUrashima, TadasuKarabagias, IoannisUrban, PhilippeKarafiloglou, PadeleimonUrbanczyk-Lipkowska, ZofiaKarakasidis, T.E.Urbano, Ana MargaridaKaramać, MagdalenaUrra, FelixKaramanoli, KaterinaUsami, YoshihideKardos, JózsefUsui, KenjiKarim, Mohammad RezaulUsuki, ToyonobuKarlovsky, PetrUsuki, YoshinosukeKårlund, AnnaUto, YoshikazuKarmakar, ParthaUygun, SahraKarnaouri, AnthiUziel, OritKarousou, EvgeniaV.G., BessergenevKarpiński, Tomasz M.Vaca, InmaculadaKasem, Kasem K.Vagner, JosefKashina, AnnaVaidya, AmitaKashiwagi, ShinichiroVaidyanathan, BharatKashman, YoelVaidyanathan, GanesanKaškonienė, VilmaVajda, SandorKasper, DennisValdes, EsperanzaKasperkiewicz, PaulinaValencia, GregorioKasten-Jolly, JaneValentao, PatriciaKataev, EvgenyValente, SergioKatahira, RuiValenti, Maria TeresaKataoka, HiroyukiValentine, Rudy J.Kataoka, NaoyukiValentová, KateřinaKataoka, RyotaValenzuela, HectorKatayama, HironoriValenzuela, RodrigoKatayama, ShigeruValero, Marta SofíaKatepalli, HariValgimigli, LucaKathuria, HimanshuValles, Soraya L.Kato, YasumasaVallet, ValérieKatogi, HideakiValletta, AlessioKatsantonis, DimitriosVallini, GiovanniKatsila, TheodoraValoppi, FabioKattamuri, PadmanabhaVamanu, EmanuelKatuchova, JanaVan Ballegooijen, HanneKaufman, Teodoro S.Van Bocxlaer, KatrienKauppinen, AnuVan Cruchten, StevenKaveh, HamedVan Dam, Jan E.G.Kavita, KumariVan Dam, R. MichaelKawabata, KyuichiVan De Sluis, A.J.A. (Bart)Kawabe, AkiraVan Den Bergh, HubertKawada, ManabuVan Der Kuyl, AntoinetteKawaguchi, Shin-ichiVan Der Meulen, Nicholas P.Kawaguchi, ToshikazuVan Dyke, Mark E.Kawakami, SusumuVan Ee, Benjamin W.Kawakita, HidetakaVan Griensven, LeoKawamura, AkiraVan Herwijnen, Hendrikus W. G.Kawamura, FumioVan Horn, WadeKawamura, IzuruVan Ree, TeunisKawao, NaoyukiVan Rijn, Richard M.Kawa-Rygielska, JoannaVanderlei, Maria De FátimaKawase, YoshinoriVanelle, PatriceKawashima, HidekiVann, WillieKawashima, YasushiVannini, AndreaKawato, TakayukiVanoni, Maria AntoniettaKawato, YujiVányolós, AttilaKay, ColinVáradi, GyörgyiKay, GraemeVáradi, JuditKayano, Shin-ichiVaradwaj, Pradeep R.Kazeto, YukinoriVaraprasad, KokkaracheduKazunori, KadotaVareda, JoãoKebede, Biniam T.Varga, ElisabethKedage, VivekanandaVarga, ZoltanKeglevich, GyörgyVaroni, Elena MariaKeiler, Annekathrin MartinaVarrot, AnnabelleKeith, Jason M.Varvounis, GeorgeKéki, SándorVasaikar, SuhasKeller, AdrianVasas, AndreaKeller, AndreasVasicek, OndrejKello, MartinVasile, FrancescaKellogg, JoshVasiljevic, TodorKelly Aparecida Dias, De Freitas CastroVasilyev, AleksanderKelly, Christopher V.Vasin, Mikhail V.Kemp, Michael G.Vasko, PetraKeplinger, TobiasVassalle, CristinaKeppler, JuliaVassilev, Nikolay G.Kerch, GarryVassilopoulos, GeorgeKerimi, AsiminaVaughan, Martha MKerr, WilliamVautier, SimonKerrigan, Nessan J.Vavitsas, KonstantinosKerrihard, Adrian L.Vaz Junior, SilvioKerwin, Sean M.Vaz, JosianaKessel, DavidVazquez Belda, BeatrizKeszler, AgnesVázquez Tato, M. PilarKetav Kulkarni, KetavVázquez, José AntonioKetkar, AmitVazquez, SantiKeum, Young-SooVazquez-Martínez, José AlfredoKevadiya, BhaveahVecino, XanelKeyzers, RobVega Gutierrez, SarathKhadka, ManojVejux, AnneKhairullina, VeronikaVelasco-Vélez, Juan JesúsKhalil, Ibrahim R.Veldhuizen, EdwinKhalil, ZeinabVeleeparambil, ManojKhallouki, FaridVelena, A.Khan, Mohammad SharifVelez, ZéliaKhan, MohsinVelkov, TonyKhan, MujeebVella, Laura JayneKhan, MushfiquddinVemula, HarikaKhan, NaghmaVenditti, AlessandroKhan, Omar F.Venkatraman, VishweshKhan, ShafiqVennerstrom, JonathanKhatri, BhuwanVenturi, FrancescaKhatri, KshitijVenus, JoachimKhattab, MuhammadVereecken, LucKhaw, KooiVerestiuc, LilianaKhisamutdinov, EmilVergara, DanieleKhlebnikov, Andrei I.Verhage, LeonieKhodanovich, MarinaVerhoeven, AdrieKhoddami, AliVerma, PragyaKhotimchenko, Maxim Y.Verma, RajniKhoury, SamarVermaas, Joshua V.Khurshid, ZohaibVermeir, LienKiani, AmirkianooshVermeulen, ChristiaanKicel, AgnieszkaVerotta, LuisellaKiczorowska, BożenaVerweij, HendrikKiec-Kononowicz, KatarzynaVetere, AmedeoKiefer, JohannesVetter, Stefan W.Kieffer, BrunoVetvicka, VaclavKieliszek, MarekVezzu, DileepKihara, Shin-ichiViapiana, AgnieszkaKijjoa, AnakeViard, MathiasKikowska, MałgorzataVicas, SimonaKikuchi, HaruhisaVicente, Manuel Sastre DeKikuchi, MacotoVichapong, JitladaKilmartin, PaulVictor, BrunoKim, BongleeVig, KomalKim, ChonsaengVíglaský, ViktorKim, Choon YoungViglianti, BenjaminKim, Chul YoungVigneron, AurelienKim, Chung SubVila, CarlosKim, ChyerVilar, JoseKim, DaehwanVilela, AliceKim, DonginVillano, DeboraKim, DongukVillaverde, Juan JoséKim, HangunVincentini, OlimpiaKim, HoonVinciguerra, ManlioKim, Hoy-TaekVining, KellyKim, HyesunVirjamo, VirpiKim, Hyeung-RakVirzonis, DariusKim, HyoungsuVisioli, FrancescoKim, Hyung SikViso, AlmaKim, Hyung-JoonVistoli, GiulioKim, HyunwooVitalini, SaraKim, InKyeomVitalone, AnnabellaKim, JaheonVladimira, TomeckovaKim, JeongkwonVladimir-Knežević, SandaKim, Ji YeonVlamos, PanayiotisKim, Joo-HwanVlashi, ErinaKim, JunheonVo, Duc-ThangKim, Keun-SikVodnar, Dan CristianKim, Ki HyunVogel, AlexanderKim, Kil-SooVoglmeir, JosefKim, KwanggiVogt, MartinKim, KyounghyunVoiniciuc, CătălinKim, Mi-hyunVolcho, Konstantin P.Kim, NamshinVoliani, ValerioKim, Ok TaeVolk, DavidKim, Sang HoonVolny, MichaelKim, Seok-JhinVolonterio, AlessandroKim, Soo UnVolpe, Maria GraziaKim, Sung-KooVon Knethen, AndreasKim, T. DoohunVondrasek, JiriKim, Tae KonVorob'ev, Mikhail M.Kim, Tae-HyungVougogiannopoulou, KonstantinaKim, YangVoutchkova-Kostal, AdelinaKim, YongaeVoziyan, PaulKim, Youn-ChulVranes, MilanKim, Yu ChulVrecl, MilkaKim, YunjeongVriend, JerryKimira, YosifumiVrontaki, EleniKimura, HidetoVrsaljko, DomagojKimura, Ken-ichiVuković, RosemaryKimura, SeisukeVullo, DanielaKimura, ShunsakuVuong, Thu V.Kinaciyan, TamarVuorela, HeikkiKinder, DavidVyazovkin, SergeyKinoshita, AyaeVynios, DemitriosKinoshita, TakayoshiVyviurska, OlgaKinthada, RamakumarWabiko, HiroetsuKintzel, Polly E.Wada, JunKinzhalov, Mikhail A.Wadati, HirokiKiparissides, CostasWade, Charles E.Kipper, KarinWagemaker, Tais Aleriana LuconKirienko, NatashaWagler, JörgKirillov, Alexander M.Wagner, AndreasKirillova, AlinaWagner, AnikaKirsch, Gilbert H.Wagner, GabrieleKirton, Stewart B.Wagner, PawelKiryukhin, Maxim V.Wahab, M. FarooqKiss, AnnaWähälä, KristiinaKiss, JánosWahl, PatrickKiss, Nóra ZsuzsaWakioka, MasayukiKitagawa, MasatoshiWaksmundzka-Hajnos, MonikaKitagishi, HiroakiWaldmeier, FelixKitahama, YasutakaWalker, HeatherKitamura, AkiraWalkinshaw, MalcolmKitano, YoshikazuWallace, DavidKitazawa, TakafumiWaller, DeborahKiuru, PaulaWall-Medrano, AbrahamKizana, EddyWallner, BjörnKjaergaard, HenrikWalsby, CharlesKlaerner, Frank-GerritWalton, JamesKlajnert-Maculewicz, BarbaraWalton, JohnKlarskov, KlausWan, ChunpengKlavina, LauraWan, EdwinKleier, Daniel A.Wan, JianboKlein, AxelWan, ShibiaoKlensporf-Pawlik, DorotaWan, XuehuaKlose, ThomasWanders, DesireeKlosterman, JeremyWandzik, IlonaKluza, JéromeWang, BiboKlyubin, IgorWang, BoKmetič, IvanaWang, Bo-ChengKnapp, SpencerWang, Chao-MinKnez, ŽeljkoWang, Chin-KunKnight, AlanWang, Chiu-YenKnight, DavidWang, CundeKnippschild, UweWang, FazuoKnittelfelder, OskarWang, Feng-ShengKnölker, Hans-JoachimWang, GuozhengKnoshaug, EricWang, HsiuyingKnudsen, Gabriel ArtherWang, Hui-Min DavidKnupp, GerdWang, JeffreyKo, Shun‑YaoWang, Jian-WenKobayakawa, TatsuWang, JianxuKobayashi, KenichiWang, JiminKobayashi, YayoiWang, JuexinKobayashi, YutaroWang, JunKobori, AkioWang, JunjieKoch, MatthiasWang, KaiKoch, WojciechWang, Lai-XiKocot, JoannaWang, LeiKodama, DaisukeWang, Li-XinKodoudakis, NikosWang, LizhuKoerner, RomanWang, MengyuKöfalvi, AttilaWang, Michael CaiKogan, MarceloWang, MingmingKoh, Cho YeowWang, PengchengKöhler, KarstenWang, QiuanKohlmann, HolgerWang, QunKohyama, NorikoWang, Richard R.-C.Koizumi, Take-akiWang, Robert Y.-L.Kojima, HideoWang, RongshengKojima-Yuasa, AkikoWang, RuibingKok, Victor C.Wang, San-LangKokado, KentaWang, ShengnianKokoška, LadislavWang, Sheng-YangKokotos, Christoforos G.Wang, Shih-WeiKokotos, GeorgeWang, Tong-HongKoksharova, OlgaWang, Tsung-JenKolaczkowski, BryanWang, Tzu-FanKolanowski, Jacek L.Wang, WentianKolaric, BrankoWang, XiaowanKolesárová, AdrianaWang, XinkunKoller, UlrichWang, XinwenKolocouris, AntoniosWang, XiumeiKomaki, HisayukiWang, XiupengKomasa, SatoshiWang, XizuKomeyama, KimihiroWang, XuewenKomilis, DimitriosWang, Yi-ChengKommineni, VallyWang, YifeiKonar, ArkaprabhaWang, YuKong Thoo Lin, PaulWang, Yun-MingKong, In-SooWang, ZenengKong, LingxueWang, ZhaohuiKong, QingkeWang, ZheKong, WeiliWang, ZhengbaoKonieczna, LucynaWang, ZhihanKönig, GerhardWang, ZhijunKönigs, Rene M.Ward, LiamKonno, HiroyukiWarnken, ZacharyKonno, KatsuhiroWaser, MarioKontziampasis, DimitriosWasylka, Justyna PłotkaKoo, ByungjinWatanabe, Hiroshi C.Kool, JWatanabe, MikiKootala, SujitWatanabe, RyuichiKopec, RachelWatanabe, ShinichiKopecký, VladimírWathes, ClaireKopel, PavelWatkins, A. NealKopjar, MirelaWatkins, Gordon LeonardKoppel, KadriWaxman, MichaelKorb, MarcusWdowin, MagdalenaKorfei, MartinaWebb, Kimberly M.Korkina, Ludmila G.Weber, CameronKornprat, PeterWeber, JohnKoroteev, Pavel S.Webster, Ruth L.Korsching, EberhardWege, StefanieKorstad, JohnWei, ChangyongKorzeniowska, MargorzataWei, CunxuKošak, AljošaWei, HuaKoschella, AndreasWei, JiaKoshino, HiroyukiWei, KongchangKoslowski, ThorstenWei, QiangKostakis, GeorgeWei, Zhao-JunKostakis, IoannisWeichert, JameyKostecka, MałgorzataWeiss, JohannaKosterin, OlegWelch, AlanKosters, AstridWelgamage Don, AakashKostiainen, MauriWeller, Michael G.Kosuge, YasuhiroWelling, MickKotek, JanWen, RongKotiuga, MicheleWeng, Ching-FengKotloski, RobertWeng, LihuiKotlowski, RomanWeng, Meng-ShihKotsikorou, EvangeliaWeng, XiaoyuKotsuchibashi, YoheiWenland, JuergenKotta-Loizou, IolyWertz, Philip W.Kotula-Balak, MalgorzataWesolowski, MarekKoufaki, MariaWest, James D.Koukounaras, AthanasiosWestcott, Stephen A.Koulen, PeterWesterlind, UlrikaKoupenova, MilkaWestmark, Cara J.Koutelidakis, Antonios E.Westwell, AndrewKoutentis, Panayiotis A.Westwell, Andrew D.Koutselas, IoannisWhelan, RebeccaKouwer, PaulWhite, Jason CKouza, MaksimWhite, PeterKovac, StjepanaWhite, Robert L.Kovács, LajosWhitty, AdrianKoval, AlexeyWhorley, Sarah B.Kovalenko, SergeyWianowska, DorotaKovinich, NikWiczk, WiesławKovvasu, SuryaWidenmeyer, MarcKowalak, StanisławWidhalm, MichaelKowalczewski, PrzemysławWie, Myung-BokKowalska, EwaWiedemann, DennisKowalska, KatarzynaWiedman, GregoryKowalska-Góralska, MonikaWientjes, EmilieKowalski, KonradWiesław, WiczkowskiKowalski, StefanjanWiesner, SilkeKoyama, TakeshiWiesner-Reinhold, MelanieKoyioni, MariaWiggins, GregKozaki, AkikoWijesinghe, Sudesh L.Kozakiewicz, AnnaWijethunga, TharangaKozieł, EdmundWilding, MatthewKoziel, Jacek A.Wilds, Christopher J.Kozlevčar, BojanWilkinson, J. M.Kozliak, Evguenii I.Willerth, Stephanie MKozlov, SergueiWilliams, Elizabeth AKozłowska, MariolaWilliams, GarethKozuch, SebastianWilliams, Ian H.Kragović, MilanWilliams, Noelle S.Krajka-Kuźniak, ViolettaWilliams, Paul JKramer-Marek, GabrielaWillner, ItamarKranjac, MarinaWills, MartinKrasniewska, KarolinaWilson, Anne M.Krchnak, ViktorWilson, DavidKregiel, DorotaWilson, Heather L.Kremleva, AlenaWilson, Justin J.Kren, VladimirWilson, ThomasKressler, JoergWilusz, JeffKries, HajoWinckler, ThomasKrisch, JuditWinkler, JohannesKristensen, MieWińska, KatarzynaKrivovichev, VladimirWitcher, AnthonyKrizbai, IstvanWitczak, ZbigniewKról, EwelinaWitorsch, Raphael JayKról-Kilińska, ŻanetaWittstock, UteKroncke, Brett MWłodarczyk, MaciejKrstin, SonjaWoelwer-Rieck, UrsulaKruger, Jacob S.Wójciak-Kosior, MagdalenaKruszynski, RafalWojciechowski, ŁukaszKrylov, Sergey N.Wojcieszak, RobertKschischo, MaikWójcik, GrzegorzKu, Kang-MoWójkowska, Dagmara WeronikaKu, SeockmoWojnárovits, LaszloKuan, Yu-HsiangWojtunik-Kulesza, Karolina A.Kuang, HuihuiWojtyczka, RobertKubala, LukášWolf, ElmarKubatka, PeterWolfe, Lisa M.Kubica, PawełWolfram, EvelynKubíček, VojtěchWolny, JuliuszKubicova, LenkaWolny, LidiaKubina, RobertWong, Chung F.Kubiński, KonradWong, Clarence T. T.Kubo, TakanoriWong, Dominic W. S.Kubota, TakaakiWong, Man-kinKuca, KamilWong, ShunKucerova, MartaWong, Vincent Kam WaiKucinska-Lipka, JustynaWong, Wai ShiuKuckling, DirkWood, DelilahKucuk, IsrafilWood, Troy D.Kucukkal, TugbaWoolbright, Benjamin L.Kuenz, AnjaWoolridge, ElisaKuerten, StefanieWöstemeyer, JohannesKues, Wilfried A.Woster, PatrickKugler, SzymonWoźniak, ŁukaszKuhlmann, MarkusWozniak-Knopp, GordanaKuimova, MarinaWrigstedt, PauliKujawska, MałgorzataWu, Chi-ReiKujawski, JacekWu, Chung-YiKukli, KaupoWu, DayongKukovec, Boris-MarkoWu, Hung-ChinKukreja, MuskanWu, Jer-HorngKukshal, VandnaWu, JiaminKukula-Koch, WirginiaWu, Jian-LinKulkarni, ShashankWu, JianmingKulma, AnnaWu, Jing-YunKumar, AmitWu, Jin-YiKumar, DhirajWu, Li-ChenKumar, KrishanWu, Ming-JiuanKumar, ManuWu, NanKumar, NarenderWu, Pao-ChuKumar, NarendraWu, Pei-FungKumar, NitinWu, Ph.D., WeiKumar, PradeepWu, Sheng-ChiKumar, PrasunWu, Shu-JuKumar, SunilWu, Shu-PaoKumari, AshaWu, SiKung, Chung-WeiWu, SonglinKunjukunju, SangeethaWu, TerenceKuno, ToshiyaWu, Tian-ShungKunz, HorstWu, Ting-FengKunz, WolframWu, TongzhiKuo, Chung-WenWu, Tzong-YuanKuo, Meng-IWu, Wen-LuanKuo, Ping-ChungWu, XiangfaKuo, Shiao-WeiWu, XinKuo, Yao-HaurWu, Xin-PingKupczyński, RobertWu, XuejianKüppers, StephanWu, YunlongKurdowska, Anna K.Wu, Yu-TseKurek, MarcinWu, ZhanhongKuroboshi, ManabuWu, ZhiqiangKuroda, ChiakiWuethrich, AlainKurtán, TiborWujec, MonicaKuś, PiotrWulff, Jeremy E.Kusch, PeterWuttke, StefanKushkevych, IvanXavier, Cristina Pinto RibeiroKuttel, MaryXavier, Nuno ManuelKuwahara, MasayasuXhaard, HenriKuyukina, Maria S.Xi, WeixianKuźnik, NikodemXia, JunfeiKuzuhara, DaikiXia, JunfengKuzuya, AkinoriXia, TianKwak, Jong HwanXiang, ChunhuiKwak, KyungwonXiang, DongxiKwak, SeonyeongXiao, DequanKwakowsky, AndreaXiao, KaijunKwan, AnnXiao, LiKwasniewska, JolantaXiao, ZhengtaoKweon, Oh-KyeongXiaoli, AlusKwiatkowska, KatarzynaXie, HongKwiatkowski, MiroslawXie, RanKwiecień, IwonaXie, WankunKwo, Hang FaiXie, WenyanKwon, Soon-HongXie, YongshuKwon, Young-WanXie, ZhigangKyle, StuartXie, Zhong-RuKynický, JindřichXin, DongyueKyzioł, KarolXin, HaiLa Barbera, GiorgiaXing, ChengguoLa Ferla, BarbaraXu, BinLa Motta, ConcettinaXu, ChunfuLa Penna, GiovanniXu, DazhongLa Russa, DanieleXu, JiminLa Spada, LuigiXu, NanLaarmann, TimXu, PengLabbadia, JohnXu, QinziLaBean, Thomas H.Xu, SuowenLabrou, NikolaosXu, YanLabruna, GiuseppeXuan, JunyuLachenmeier, DirkXuan, Tran DangLachman, JaromirYabuki, SoichiLachowicz, Joanna IzabelaYadav, DhananjayLachowicz, SabinaYamada, HiroyukiLafarga, TomasYamada, KojiLagana, AldoYamada, ShuheiLago, João Henrique GhilardiYamagishi, TakehiroLahooti, HooshangYamaguchi, KanjiLai, QinghuaYamaguchi, MakotoLai, YandongYamaguchi, MasahikoLaird, EamonYamaki, KouyaLaity, Peter RYamamoto, YasunoriLakard, BorisYamashita, AtsushiLalas, StavrosYamashita, TeruhitoLallana, EnriqueYamato, TakehikoLam, Kim-HungYamauchi, SatoshiLam, Kwok HoYan, BowenLambert, JoshuaYan, DayunLambert, Nevin A.Yan, DongqingLambrinidis, GeorgeYan, JinLamzin, VictorYan, JinhuiLan, LanYan, ShengminLan, WenjianYan, XuehaiLan, YuYanai, HikaruLan, YuchengYanase, EmikoLanças, FernandoYancheva, Denitsa Y.Lancelin, Jean-marcYanda, MuraliLandi, MarcoYandeau-Nelson, Marna D.Lang, SiegmundYang, Chao-HsunLange, HeikoYang, Chia-NingLangendorf, ChristopherYang, Chun-LongLanger, Jerzy JYang, Ding-YahLanger, ThierryYang, Feng-LingLangford, T. DianneYang, HongshunLannoy, DamienYang, InhoLanzerstorfer, PeterYang, KunLappan, UweYang, LijiangLaranjinha, João AntonioYang, Seo YoungLara-Sanchez, AgustinYang, Seung HwanLarobina, DomenicoYang, Shao-YuLaronze-Cochard, MarieYang, Sze-Ming MildredLarraneta Landa, EnekoYang, TaoLarson, Nicholas B.Yang, Teng-ChunLash, TimothyYang, Wei-hsiungŁatka, LeszekYang, XinmaiLatosińska, Jolanta NataliaYang, XiuweiLatouche, CamilleYang, YongkangLattanzio, RossanoYang, ZhiboLau, Christopher S.Yannakopoulou, KonstantinaLaube, MarkusYao, JiayiLaunay, Jean MarieYao, WeirongLaurent, FerriéYao, YangLaurenti, EnzoYao, ZhiliLauro, Maria RosariaYaoita, YasunoriLauschke, VolkerYaremenko, Ivan A.Laustsen, Andreas HougaardYasir, IqbalLavandera, José LuisYasuda, ShinLavelli, VeraYasuda, TakeshiLavilla, RodolfoYasui, KyuichiLavoie, SergeYates, EdwinLázár, IstvánYates, Edwin AlexanderLazarus, KyrenYates, Matthew Z.Lazzarato, LorettaYazdan Panah, NimaLazzari, ClaudioYe, EnyiLazzaroni, RobertoYe, HuaiyuLe Borgne, MarcYe, ZhouLe Bourvellec, CarineYeh, Jan-yingLe Ouay, BenjaminYeh, Jwu-LaiLe Questel, Jean-yvesYeh, Shu-LanLe Roes-Hill, MarilizeYen, Chia-HungLe, Hoang-ThanhYen, Feng-LinLeadbeater, NicholasYen, TimothyLeal, Ana MendesYeo, SynLear, Benjamin J.Yerabolu, Jayasudhan ReddyLebar, MatthewYi, MyunggiLebeau, LucYi, Tae HooLebedev, NikolaiYi, TaoLeblebici, Mumin EnisYi, Young-SuLecaille, FabienYiannakopoulou, EugeniaLeclercq, GuyYin, ShengLeclercq, LoïcYlijoki, Kai E. O.Lecomte, PhilippeYokohira, MasanaoLederkremer, Gerardo Z.Yokomatsu, TsutomuLedo, AnaYokoshima, SatoshiLedwon, PrzemyslawYokota, KazushigeLee, Bong HoYokota, Shin-ichiLee, Bor-ShiunnYokota, ShinsoLee, ChangwonYokoya, MasashiLee, Che-HsinYokoyama, ShinjiLee, Cheng-IYonemochi, EtsuoLee, Chen-YuYoneshiro, TakeshiLee, Chia-HungYoo, DongwonLee, Ching-KuoYoo, Ki-OugLee, E-jianYoon, Do-YoungLee, GihyunYoon, HyeonseokLee, GyoYoon, In-SooLee, Hian KeeYoon, JinhwanLee, Hong InYoon, Kee-DongLee, Huei-JaneYoon, Moon-YoungLee, Hye SukYoshida, HiderouLee, HyunbokYoshida, KazunariLee, IlkeunYoshida, KumiLee, Jae WookYoshida, Nidia CristianeLee, JaehwiYoshikawa, YutakaLee, Jae-SukYoshimura, MorioLee, Jin-KooYoshimura, YuichiLee, Jin-KyunYoshinari, NobutoLee, Jiun-hawYoshitaka, YamaguchiLee, JongsungYoshiya, TakuLee, Joo HyoungYoza, Brandon A.Lee, Jun SeokYu, Cheng-JuLee, Jun SikYu, ChenglongLee, Keun-HyeungYu, GuoqinLee, Kwang HoYu, HongweiLee, Meng-JenYu, HuaLee, Meng-shiouYu, Jae WoongLee, Mi KyeongYu, JiayiLee, Min WonYu, LinlongLee, Sang GilYu, MeifangLee, Sang-HanYu, MeihuaLee, SanghyeobYu, MinzhongLee, SangkilYu, RongminLee, Sang-MyungYu, WenquanLee, Seok JaeYu, Yan PingLee, Shih-yuYuan, Min-HaoLee, Soo YoungYuan, ShinshengLee, Sung HoYuan, YaxiaLee, T. RandallYuan, ZhihongLee, Tai-LinYuan, ZhiqinLee, Yeon-JuYuan-Bin, ChengLee, Yong RokYue, PatrickLee, ZhenghongYue, YanfengLefebvre, FredericYukl, Erik T.Lefebvre, TonyYun, HyungdonLefkimmiatis, KonstantinosYusa, Shin-ichiLegentil, LaurentZabielska-Koczywąs, KatarzynaLéger, BastienZaccheroni, NelsiLegrand, François XavierZacconi, Flavia C.Lehmann, HelmarZacharias, MartinLehner, MałgorzataZacharis, Constantinos K.Lehotay, Steven J.Zacharof, MyrtoLei, FanZaferanloo, BitaLein, MatthiasZagotto, GiuseppeLeitão, AnaZaharia, ValentinLeitão, Jorge H.Zahid, MalihaLeite Sampaio, BrunoZaid, HilalLekka, MalgorzataZaidi, SaheemLemeune, AllaZaitone, Sawsan A.Lemli, BeátaZajdel, PawełLemoine, PierricZakrevsky, PaulLemon, ChristopherZakrzewski, JerzyLemos, Manuel L.Zalc, BernardLen, ChristopheZaleśny, RobertLenardão, Eder J.Zalubovskis, RaivisLenart, AndrzejZamaratskaia, GaliaLentini, GiovanniZambrowicz, AleksandraLenzen, SigurdZamora Marin, FernandoLeón, JosefaZamudio-Rivera, Luis S.Leon, Juan FranciscoZamyatnin, Jr., Andrey A.Leonard, StephenZandarashvili, LevaniLeong, Max K.Zandi, RoyaLeonidas, Demetres D.Zapico, Sara C.Leonidas, PalilisZappia, GiovanniLeonzio, GraziaZappia, StefaniaLepenies, BerndZaprutko, LucjuszLeporatti, StefanoZaręba, Jan K.Lepore, MariaZarejousheghani, MashaalahLeroy, BaptisteZarra, IgnacioLesner, AdamZarrelli, ArmandoLesniewska, EricZarzycki, Pawel K.Lessard, BenoitZastre, JasonLesyk, Roman B.Zdarta, JakubLeung, Chung-HangZeiner, MichaelaLeung, IvanhoeZeitler, AxelLeung, Ka HoZeković, ZoranLeung, Yuk-manZeleny, ReinhardLewandowicz, GrażynaZelkó, RománaLewandowski, MikołajZeng, GuangmingLewinska, AnnaZeng, HaishanLewis, KarenZeng, HuaqiangLewis, KathyZeng, ShuwenLewis, RandolphŻesławska, EwaLewkowicz, ElizabethZgórka, GrażynaLewkowski, Jarosław A.Zha, ShangwenLey, Alexandra C.Zhang, BinLeymarie, OlivierZhang, BoLézot, FrédéricZhang, DimingLi, AilinZhang, DuoLi, ChengZhang, FanLi, ChunshunZhang, FumingLi, DegangZhang, GangLi, Fu-shuangZhang, HengyouLi, GuohuiZhang, HongqiaoLi, Hou-JinZhang, HuLi, Hua-BinZhang, HuaLi, JieZhang, HuaweiLi, JinZhang, JianyingLi, JinboZhang, JieLi, JinghaoZhang, JingjingLi, KaichangZhang, JinnanLi, Kuo-BinZhang, JunboLi, Mei-LingZhang, JunranLi, MiaomiaoZhang, JunzengLi, MingqiangZhang, KaiLi, Na (China)Zhang, KeLi, Na (Macao, China)Zhang, MingLi, NingyangZhang, PangzhenLi, PingZhang, PengLi, ShaoZhang, QianqianLi, Shao-GangZhang, QichunLi, ShujunZhang, Qing-WenLi, SihengZhang, QiongLi, TianhuZhang, RuiyongLi, WeiZhang, RunLi, Wen-WuZhang, ShaozhongLi, WenyiZhang, TaoLi, Xiang-GuoZhang, WeiLi, XiaohuaZhang, WenhengLi, XiaopengZhang, WujieLi, XinghuiZhang, XiaomingLi, Yan (China)Zhang, XuanjunLi, Yan (New Zealand)Zhang, Xueqiang AlexLi, Yan (Singapore)Zhang, XueqingLi, YangZhang, YaolinLi, YanmeiZhang, YingyueLi, Yan-MeiZhang, YixiangLi, YifanZhang, YixuanLi, YinZhang, YuLi, YingZhang, YunkaiLi, YiwenZhang, ZhaoweiLi, Zhi-MinhgZhang, ZhenbinLi, ZhonghuZhang, Zhong-YinLi, ZijianZhao, Jin-HaoLiagre, BertrandZhao, LiangLiang, KangZhao, RuimingLiang, YuerongZhao, ShanLiang, ZhibinZhao, XiaoaiLiao, Jyh-feiZhao, XinxinLiao, Pei-ChunZhao, YuxiaLiao, Shu-ChuanZhao, ZongminLiao, WupengZhbannikov, IlyaLiao, Yi-JenZhdanov, Vladimir P.Liberto, EricaZheng, LiqiuLicciardello, GraziaZheng, ShilongLicea Navaro, Alexei FedorovishZheng, XiaolongLiddelow, ShaneZheng, XintingLidon, FernandoZheng, ZhaoliangLikozar, BlažZhong, HaizhenLim, Beong OuZhong, TuhuaLim, Dong ChanZhong, Wei-ZhuLim, Ji-HongZhou, BangjunLim, JunxianZhou, Carol L. EcaleLim, YoonghoZhou, DezhongLima, Marcos Dos SantosZhou, Guo-PingLima-Neto, BeneditoZhou, HaibingLin, Chia-HerZhou, JiaLin, Chih-ChienZhou, LiliLin, Chih-LiZhou, ShengbinLin, Ching-HsuanZhou, ShuanhuLin, Chiu-YueZhou, XiaoshunLin, Chung-HoZhou, ZheLin, ErickZhu, ChangfuLin, GuitingZhu, FengLin, Hai-ShuZhu, LiangdongLin, Jung-ChunZhu, ShanLin, Kuo-ShyanZhu, WeiLin, Liang-TzungZhu, XiaoshanLin, LigenZhu, YingLin, Long-LiuZhu, ZhenweiLin, Mei-HsiangZhukov, IgorLin, Mei-hueyZhukova, NataliaLin, Shih-YiŽiarovská, JanaLin, Shwu-jiuanZiegler, JoergLin, Shyh-HsiangZiegler, ThomasLin, Tzu-EnZiegler-Borowska, MartaLin, XiaojieZielinska, BeataLin, Yu-ShenZielińska, SylwiaLin, ZekaiZielińska-Jurek, AnnaLin, ZhoumengZielińska-Pisklak, Monika A.Lin, ZongtaoZierkiewicz, WiktorLincoln, PerZimmer, ElizabethLindemann, PeterZimoch-Korzycka, AnnaLindequist, UlrikeZinchenko, AnatolyLindner, RobertZingales, SarahLindsey-Boltz, LauraZingue, StéphaneLinet, WolfgangZinna, FrancescoLing, MauriceZiora, ZytaLinscheid, Michael W.Zitko, JanLionetti, LillàZivcak, MarekLiou, Ying-mingZloh, MireLisciani, SilviaZlotek, UrszulaLitinas, KonstantinosZollo, MassimoLitvić, MladenŻołnowska, BeataLiu, BaiquanZolotoukhina, TatianaLiu, Cheuk LunZoltan-Istvan, SzaboLiu, CongZorc, BrancaLiu, DelongZorc, BrankaLiu, Dong-hongZotin, Alexey A.Liu, GangZou, GangLiu, GuangleiZou, PingLiu, Guang-YawZou, QuanLiu, GuoshiZovko Končić, MarijanaLiu, Hai-yangZsuzsanna, BőszeLiu, HongZubkova, OlgaLiu, Hui-kangZucca, PaoloLiu, HuolongZuluaga, PaolaLiu, JieminZuo, RanLiu, JuewenZuo, YuegangLiu, JunZupko, IstvanLiu, Jun-JenZuriaga, ElenaLiu, LeiZuvela, PetarLiu, QiangZych, MariaLiu, Qing-SongZygmunt, MagorzataLiu, Shing-HwaZygmunt, MałgorzataLiu, Shiuh-TzungŻymańczyk-Duda, EwaLiu, WensheŻyżelewicz, DorotaLiu, Xiaoxi (USA)


